# VAV2 is required for DNA repair and implicated in cancer radiotherapy resistance

**DOI:** 10.1038/s41392-021-00735-9

**Published:** 2021-08-30

**Authors:** Weiling Liu, Chuanwang Miao, Shaosen Zhang, Yachen Liu, Xiangjie Niu, Yiyi Xi, Wenjia Guo, Jiahui Chu, Ai Lin, Hongjin Liu, Xinyu Yang, Xinjie Chen, Ce Zhong, Yuling Ma, Yuqian Wang, Shihao Zhu, Shuning Liu, Wen Tan, Dongxin Lin, Chen Wu

**Affiliations:** 1 Department of Etiology and Carcinogenesis, National Cancer Center/National Clinical Research Center/Cancer Hospital, Chinese Academy of Medical Sciences and Peking Union Medical College, Beijing, China; 2 Cancer Institute, Affiliated Cancer Hospital of Xinjiang Medical University, Urumqi, China; 3 Key Laboratory of Oncology of Xinjiang Uygur Autonomous Region, Urumqi, China; 4 Department of pharmacy, Qilu Hospital, Cheeloo College of Medicine, Shandong University, Jinan, China; 5 Sun Yat-sen University Cancer Center, State Key Laboratory of Oncology in South China, Guangzhou, China; 6 Jiangsu Key Laboratory of Cancer Biomarkers, Prevention and Treatment, Collaborative Center for Cancer Personalized Medicine, Nanjing Medical University, Nanjing, China; 7 CAMS Key Laboratory of Genetics and Genomic Biology, Chinese Academy of Medical Sciences and Peking Union Medical College, Beijing, China

**Keywords:** Tumour biomarkers, Gastrointestinal cancer, Oncogenes, Predictive markers

## Abstract

Radiotherapy remains the mainstay for treatment of various types of human cancer; however, the clinical efficacy is often limited by radioresistance, in which the underlying mechanism is largely unknown. Here, using esophageal squamous cell carcinoma (ESCC) as a model, we demonstrate that guanine nucleotide exchange factor 2 (VAV2), which is overexpressed in most human cancers, plays an important role in primary and secondary radioresistance. We have discovered for the first time that VAV2 is required for the Ku70/Ku80 complex formation and participates in non-homologous end joining repair of DNA damages caused by ionizing radiation. We show that VAV2 overexpression substantially upregulates signal transducer and activator of transcription 1 (STAT1) and the STAT1 inhibitor Fludarabine can significantly promote the sensitivity of radioresistant patient-derived ESCC xenografts in vivo in mice to radiotherapy. These results shed new light on the mechanism of cancer radioresistance, which may be important for improving clinical radiotherapy.

## Introduction

Resistance to radiotherapy is one of the well-known hallmarks of cancer. The genomic and/or epigenomic alterations in tumor cells can cause primary radioresistance; but radiotherapy itself may result in secondary radioresistance, which is also mainly attributable to the genomic changes in tumor cells exposed to radiation.^1–3^ Although the alterations attributable to radioresistance in the radioresistant tumor genome have not been fully identified, aberrantly elevated DNA repair capacity is believed to play an important role. It is well-known that the major biological effect of radiotherapy in killing rapidly proliferating cancer cells is to cause DNA single-strand breaks (SSBs) and double-strand breaks (DSBs).^4,5^ In human cells, DSBs are mainly repaired by homologous recombination and non-homologous end joining (NHEJ), with the latter being predominant. It has been shown that genomic structural alterations such as DSBs and chromothripsis, the pervasive events in most types of cancer,^6,7^ can induce DNA NHEJ repair.^8^ These findings suggest that for survival and progression, most cancer cells may equip with a high activity of NHEJ repair system, which may also confer them radioresistant.

Esophageal squamous-cell carcinoma (ESCC), the most prevalent form of esophageal cancer worldwide, has poor survival rates due to the lack of typical clinical manifestations in the early stage and very limited effective therapies for the disease in the late stage.^9–11^ chemoradiotherapy is currently the main treatment regimen for patients with locally advanced or unresectable ESCC, which can improve patient survival time if the adverse events are acceptable and manageable.^12,13^ Unfortunately, the efficacy of radiotherapy for ESCC is modest and disparate in patients due to the radioresistance of tumor cells. However, why there is high proportion of ESCC that resists to radiotherapy remains poorly characterized^14^ and it is particularly important to explore what are changed in the ESCC cancer genome in terms of DNA repair system that contributes to radioresistance. Thus, it appears that ESCC is a good example for investigating the mechanism of radioresistance, which might help to develop more effective and precision cancer radiotherapy.

In our previous whole-genome sequencing study on 94 ESCC samples, we have identified 23 focal recurrent copy-number gain regions containing 1591 genes and the matched mRNA expression data showed 149 copy-number gain genes are overexpressed in tumor compared with adjacent normal samples.^15^ Recently, we have examined the functional roles of these 149 genes in ESCC cell lines using a siRNA library-based high-content screening approach and found that silencing of 18 genes had significant inhibitory effects on ESCC cell malignant phenotypes, including enhancing the sensitivity to DNA damaging chemotherapeutic agents.^16^ Since these genes are all located in the amplified chromosomal regions, it is reasonable to hypothesize that they might also be involved in primary resistance of ESCC to radiotherapy.

In the present study, we treated patient-derived xenografts (PDXs) and ESCC cells (PDCs) with ionizing radiation (IR) to identify differential genes whose aberrant expression may cause radioresistance. The radioresistance-associated genes were then integratedly analyzed with our previous whole-genome sequencing data of ESCC to seek for those are not only amplified or overexpressed but also correlated with radioresistance in ESCC. Based on this examination, guanine nucleotide exchange factor 2 (VAV2) has been identified to be critical in both primary and secondary radioresistance of ESCC cells. We have revealed for the first time that VAV2 is required for the formation of Ku70/Ku80 complex participated in DNA NHEJ repair; VAV2 upregulation significantly promotes the complex activity and thus reduces IR-induced DNA DSBs. In addition, we have demonstrated that VAV2 overexpression may cause the activation of STAT1 signaling in response to IR and inhibition of STAT1 activity by Fludarabine significantly reverses the vulnerability of ESCC cells to IR in vitro and in vivo in mouse xenograft models, suggesting that the VAV2-STAT1 axis is a target for improving radiotherapy. Furthermore, we have demonstrated that the VAV2 expression level in tumor is a potential biomarker for predicting radiosensitivity.

## Results

### Discovery of *VAV2* as a radioresistant oncogene in ESCC

**Fig. 1:**
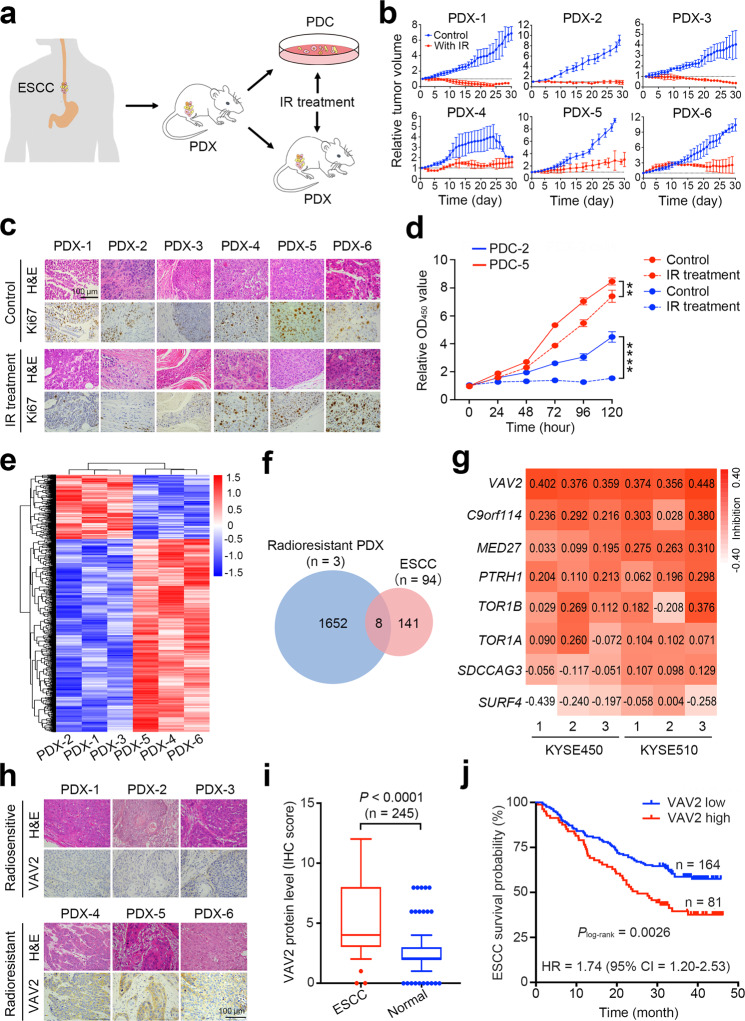
VAV2 functions as a radioresistant oncogene in ESCC. **a** Schematic diagram for the constructions of patient-derived xenograft (PDX) of esophageal squamous-cell carcinoma (ESCC), PDX-derived cells (PDC) and irradiation (IR) treatment. **b** Growth curves of PDXs from 6 individuals with ESCC, showing different sensitivity to IR. Data at each time point are mean ± SD from 3 mice. **c** Hematoxylin-eosin (H&E) and immunohistochemical (IHC) staining of Ki67 in PDX tumors with or without IR. Scale bars, 100 µm. **d** Proliferation curves of PDC-2 and PDC-5 cells with or without IR (4 Gy), showing different sensitivity to IR. Data are mean ± SEM from 3 independent experiments and most error bars are with the symbols. **P < 0.01 and ****P < 0.0001 of Student’s *t*-test. **e** Heat map showing th**e** differentially expressed genes detected by RNA-sequencing in radiosensitive or radioresistant PDXs. **f** Venn diagram displaying the overlapped genes among 1,660 apparently overexpressed genes in 3 radioresistant PDXs and 149 amplified and overexpressed genes in 94 clinical ESCC specimens as described previously.^16^ **g** Heat map displaying the inhibitory effects of silencing 8 genes on the malignant phenotypes of two ESCC cell lines. The number presents the inhibitory efficiency and each cell line had 3 independent replications. **h** H&E and IHC analysis of VAV2 protein level in radiosensitive or radioresistant PDXs. Scale bars, 100 µm. **i** Box and bar plots comparing the VAV2 protein levels between ESCC and non-tumor tissue samples. *P* of Mann–Whitney test. **j** Kaplan–Meier curves of patient survival according to the VAV2 IHC score in tumors. High, IHC score > 6 and low, IHC score ≤ 6. Also present with Kaplan–Meier plot is the hazard ratio (HR) and 95% confidence interval (CI) from multivariate Cox proportional hazard models, including age, sex, tumor stage as covariates

We first established PDX mouse models with ESCC tumors from 6 patients to seek for genes involved in radioresistance (Fig. 1a). Mice with PDX derived from each patient were divided into two groups and treated with or without IR. We found that although IR treatment inhibited the growth of all PDXs, PDXs from 3 ESCC patients (PDX-4, PDX-5 and PDX-6) had relative tumor volume (RTV) higher than 1 after IR, suggesting that these ESCC tumors were relatively radioresistant^17^ (Fig. 1b and Supplementary Table S1). Histopathological examination showed that after IR treatment, sensitive PDXs contained only small portion of ESCC cells; however, resistant PDXs remained substantially more cancer cells, with the amount of Ki67-positive cancer cells being significantly higher in resistant PDXs than that in sensitive PDXs (Fig. 1c and Supplementary Fig. S1a). Primary ESCC cells derived from radiation-sensitive or radiation-resistant PDX (PDCs) also showed differential response to IR treatment as did in vivo in PDXs (Fig. 1d).

We parallelly performed RNA sequencing of these 6 ESCC samples without IR and found that the genome-wide expression matrix could be clustered into two groups that were coincident with the grouping of radiation sensitivity (Supplementary Fig. S1b), indicating that the radioresistant determinants may be within these molecules. Differential expression analysis showed that 2212 genes were differentially expressed between radiosensitive and radioresistant ESCC (fold-change > 1.3 or <0.7, *P* < 0.05; Fig. 1e). Of 1660 genes apparently overexpressed in the 3 radioresistant PDXs, 8 genes were among our previously identified 149 amplified and overexpressed genes in 94 ESCC compared with normal tissues^16^ (Fig. 1f). Silencing the expression of each of these 8 genes suppressed the malignant phenotypes of ESCC cells in various degree and, among them, *VAV2* was the most effective (Fig. 1g). We found that radioresistant PDXs (*n* = 3) had higher VAV2 expression levels than radiosensitive PDXs (*n* = 3) (Fig. 1h) and among the investigated 245 ESCC patients, 81 (33.1%) had VAV2 overexpression in tumor compared with normal tissue as examined by immunohistochemical staining (Fig. 1i and Supplementary Fig. S1c), which was also correlated with their survival time, with the hazard ratio of death for high VAV2 level being 1.74 (95% CI = 1.20−2.53; Fig. 1j and Supplementary Table S2). These results indicate that VAV2 might play a role in ESCC radioresistance.

### IR induces ESCC cell radioresistance by evoking aberrant VAV2 overexpression

**Fig. 2:**
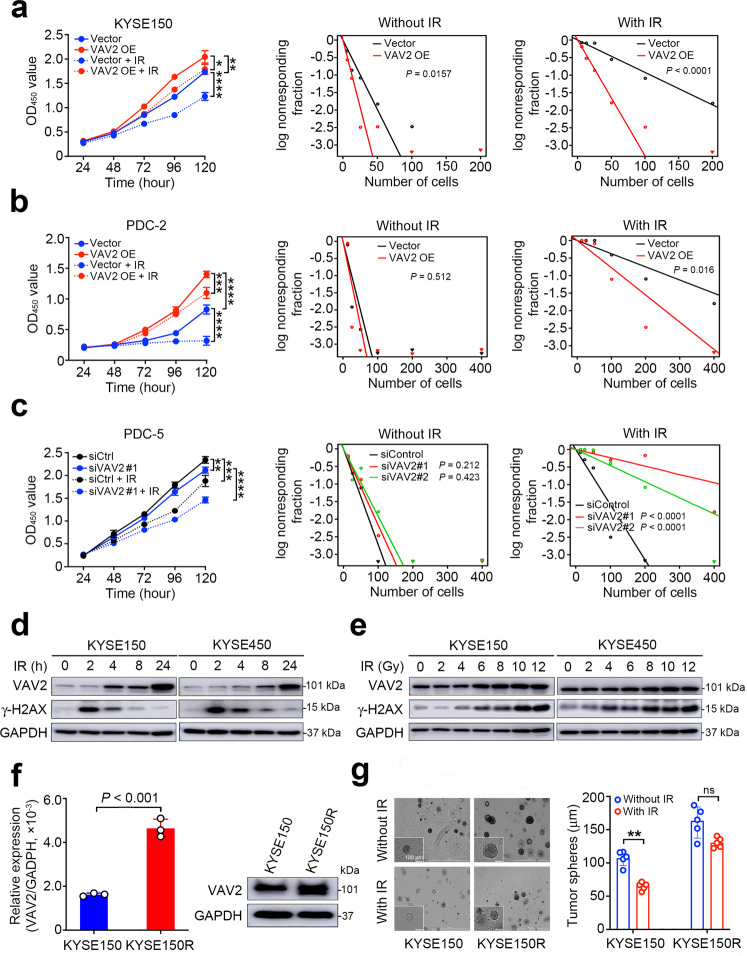
Irradiation induces ESCC cell radioresistance by evoking aberrant VAV2 overexpression. **a**, **b** Forced *VAV2* overexpression in KYSE150 cells (**a**) or radiosensitive PDC-2 cells (**b**) caused resistance of cells to IR treatment. *Left panels* show proliferation curves of cells and *right panels* shows fractions of cell survival by limiting dilution assays. IR, irradiation (4 Gy). **c** Silencing *VAV2* expression by siRNA in radioresistant PDC-5 cells significantly increased sensitivity of cells to IR treatment. *Left panels* show proliferation curves of cells with siRNA#1 (the results of siRNA#2 are shown in Supplementary Fig. S2g); *middle and right panels* show fractions of cell survival by limiting dilution assays. IR, irradiation (4 Gy). **d**, **e** Western blot analysis showed overexpression of VAV2 and γ-H2AX in ESCC cells treated with radiation (10 Gy), which is time- (**d**) and dose-dependent (**e**). **f** Comparison of VAV2 mRNA (*left panel*) and protein (*right panel*) levels in IR-induced radioresistant KYSE150 cells (KYSE150R) and its parental KYSE150 cells. Results are mean ± SEM from three independent determinations and each had three triplicates. *P* of Mann–Whitney test. **g** Tumor spheres of KYSE150 and KYSE150R cells treated with or without radiation (4 Gy). *Left panel* shows representative images of tumor spheres and *right panel* shows statistics. Scales bars, 100 μm. Data are mean ± SEM from at least three independent experiments and five fields were randomly selected for each experiment. *P < 0.05; **P < 0.01; ***P < 0.001 and ****P < 0.0001 of Student’s *t*-test

We performed the gene set enrichment analysis of 1660 genes apparently overexpressed in radioresistant PDXs and the results showed they were enriched in several pathways such as the epithelial mesenchymal transition, ultraviolet response, and protein secretion (Supplementary Fig. S2a). Interestingly, *VAV2* is within the ultraviolet response pathway (Supplementary Fig. S2b), suggesting that this gene may be involved in response to IR. To test this notion, we treated ESCC cell lines or radiosensitive primary ESCC cells (PDC-2 from PDX-2) having ectopic *VAV2* overexpression and radioresistant primary ESCC cells (PDC-5 from PDX-5) having forced *VAV2* silence with IR and found that cells overexpressing *VAV2* were not or much less sensitive to IR compared with cells without *VAV2* overexpression (Fig. 2a, b and Supplementary Fig. S2c–f); in contrast, silencing *VAV2* expression in primary radioresistant ESCC cells re-conferred sensitivity to IR (Fig. 2c and Supplementary Fig. S2g, h). Since IR can trigger a series of cellular DNA damage responses including the expression of related genes,^18–20^ we therefore treated ESCC cell lines with IR and then measure the VAV2 protein levels in different time after treatment. Cellular DNA damages were monitored using γ-H2AX, a well-known marker of DNA DSBs. We found that the VAV2 levels increased but γ-H2AX levels decreased from 2 to 24 h after cells were exposed to irradiation (Fig. 2d) and the increase in the VAV2 levels was in an irradiation dose-dependent manner (Fig. 2e). These results support our notion that VAV2 may be a DNA repair player. We then established a radioresistant ESCC cell line by repeatedly exposure of KYSE150 cells to 2 Gy IR each time until reaching the total dose of 60 Gy. We found that the resultant cell line passed for 10 passages after IR had significantly increased *VAV2* mRNA and protein expression levels compared with the paternal cell line (Fig. 2f). In parallel, the growth and tumor spheres formation abilities of the resistant cell line were significantly higher than that of the paternal cell line with or without IR treatment (Fig. 2g and Supplementary Fig. S2i–k). Collectively, these results show that VAV2 is an important molecule mediating resistance of ESCC cells to IR. We also verified the mRNA expression levels of the identified 8 genes in KYSE150R and KYSE450R cells and compared them with those in KYSE150 and KYSE450 cells without IR, respectively. The results showed that the expression levels of *MED27, PTRH1, TOR1B* and *SDCCAG3* were upregulated in both KYSE150R and KYSE450R cells while *C9orf114*, *TOR1A* and *SURF4* were upregulated in KYSE450R cells (data not shown). These results were in general consistent with the finding that these 8 genes are associated with radioresistance of ESCC.

### *VAV2* overexpression promotes DNA repair in ESCC cells

**Fig. 3:**
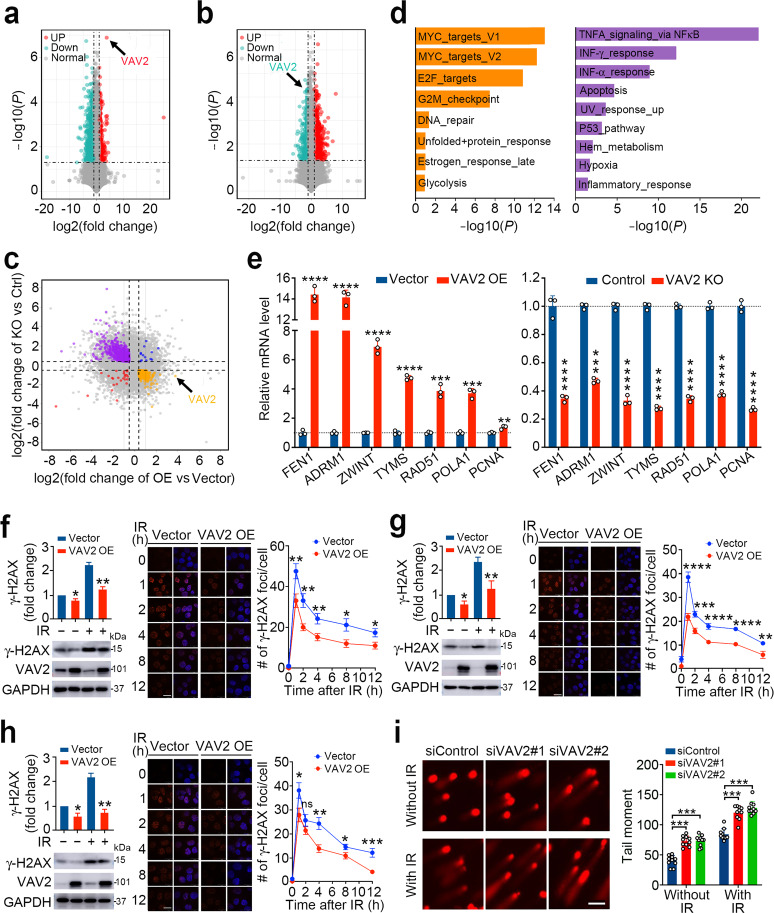
VAV2 overexpression promotes DNA repair in ESCC cells. **a**, **b** Volcano plots displaying upregulated (fold-change > 1.3; *P* < 0.05) or downregulated genes (fold-change < 0.7; *P* < 0.05) in KYSE150 cells with *VAV2* overexpression (**a**) or knockout (**b**). Data are from three experiments. **c** Volcano plot of gene expression changes in both cell lines with *VAV2* overexpression (OE) or knockout (KO). Orange, genes upregulated in cells with *VAV2* OE and downregulated in cells with *VAV2* KO. Purple, genes downregulated in cells with *VAV2* OE and upregulated and in cells with *VAV2* KO. Blue, genes upregulated in cells with *VAV2* OE or KO. Red, genes downregulated in cells with *VAV2* OE or KO. Gray, genes unchanged in cells with *VAV2* OE or KO. All *P* < 0.05 except for genes in gray color. **d** Gene set enrichment analysis (GSEA) of genes in orange or purple in **c** showed several pathways including DNA repair-related pathway positively (*left panel*) or negatively (*right panel*) correlated with *VAV2* expression. **e** Verification of the expression changes of genes in DNA repair pathway identified by GSEA in (**b**) in cells with *VAV2* OE or KO. The level of mRNA was determined by real-time-quantitative PCR and data are mean ± SEM from three independent determinations. **P < 0.01; ***P < 0.001 and ****P < 0.0001 of Student’s *t*-test. **f**–**h** DNA double-strand breaks expressed by γ-H2AX level in VAV2-overexpressing KYSE150 (**f**), KYSE450 (**g**), and KYSE30 (**h**) cells treated with or without irradiation (IR, 4 Gy). *Left panels* show γ-H2AX and VAV2 levels by western blot analysis in cells 2 h after IR. *Middle panels* show images of γ-H2AX foci in cells at various time points of IR as indicated. Scale bars, 20 µm. *Right panels* represent the statistics. Data are means ± SEM from three experiments. *P < 0.05; **P < 0.01; ***P < 0.001; ****P < 0.0001 and ns, not significant of Student’s *t*-test. **i** DNA double-strand breaks detected by comet assays in PDC-5 cells with or without *VAV2* silenced by siRNA and treated with or without IR (4 Gy). *Left panel* shows fluorescence images of comet assays. Scale bars, 100 µm. *Right panel* shows the statistics. Data are mean ± SEM from three replicates and ten fields were randomly selected for each experiment. ***P < 0.001 of Student’s *t*-test

We next explored the radioresistant mechanism of VAV2 overexpression. RNA sequencing revealed several upregulated (fold-change > 1.3, *P* < 0.05) or downregulated (fold-change < 0.7, *P* < 0.05) gene expression in ESCC cells with *VAV2* overexpression or silence (Fig. 3a, b and Supplementary Fig. S3a, b). We found that the cell proliferation and cell cycle related pathways were upregulated, but apoptosis related pathways were repressed in *VAV2*-overexpressing cells; however, in cells with *VAV2* expression knockout, the opposite results were seen (Supplementary Fig. S3c–f). We identified two sets of genes that were positively (Set 1) or negatively (Set 2) correlated with *VAV2* expression level in cells with *VAV2* overexpression or knockout (Fig. 3c and Supplementary Table S3). Gene set enrichment analysis revealed that genes in Set 1 were enriched in the MYC targets, E2F targets, G2/M checkpoint and DNA repair pathways while genes in Set 2 were enriched in the TNFα, INF response, apoptosis, UV response and P53 pathways (Fig. 3d). Since *VAV2* overexpression induces radioresistance, we therefore focused on the potential function of *VAV2* in DNA damage response. It has been known that during DNA DSBs, the G2/M checkpoint pathway is upregulated to block the cell cycle in the M phase to promote repair.^21^ The expression status of key genes in the DNA repair and G2/M checkpoint pathways were confirmed by using RT-qPCR approach showing that in cells with *VAV2* overexpression these genes were significantly upregulated, but they were significantly downregulated in cells with *VAV2* knockout (Fig. 3e and Supplementary Fig. S3g).

We then examined the role of *VAV2* in IR-induced DNA damage repair based on the above suggestive results that *VAV2* overexpression may promote DNA repair function. We found that *VAV2* overexpression significantly reduced both spontaneous and IR-induced DNA damages in ESCC cells as indicated by γ-H2AX levels analyzed by Western blotting and immunofluorescence (Fig. 3f–h); in contrast, knockout of *VAV2* significantly increased γ-H2AX levels in cells with or without IR (Supplementary Fig. S3h–j). DNA comet assays with primary ESCC cells PDC-4 and PDC-5 showed that *VAV2* silence by siRNA resulted in significantly more spontaneous and IR-induced DNA damages compared with siControl (Fig. 3i and Supplementary Fig. S3k). These results demonstrate that *VAV2* plays an important role in DNA repair, which may be the underlying mechanism for radioresistance of ESCC having *VAV2* overexpression.

### VAV2 participates in Ku70 and Ku80-mediated DNA NHEJ repair

**Fig. 4:**
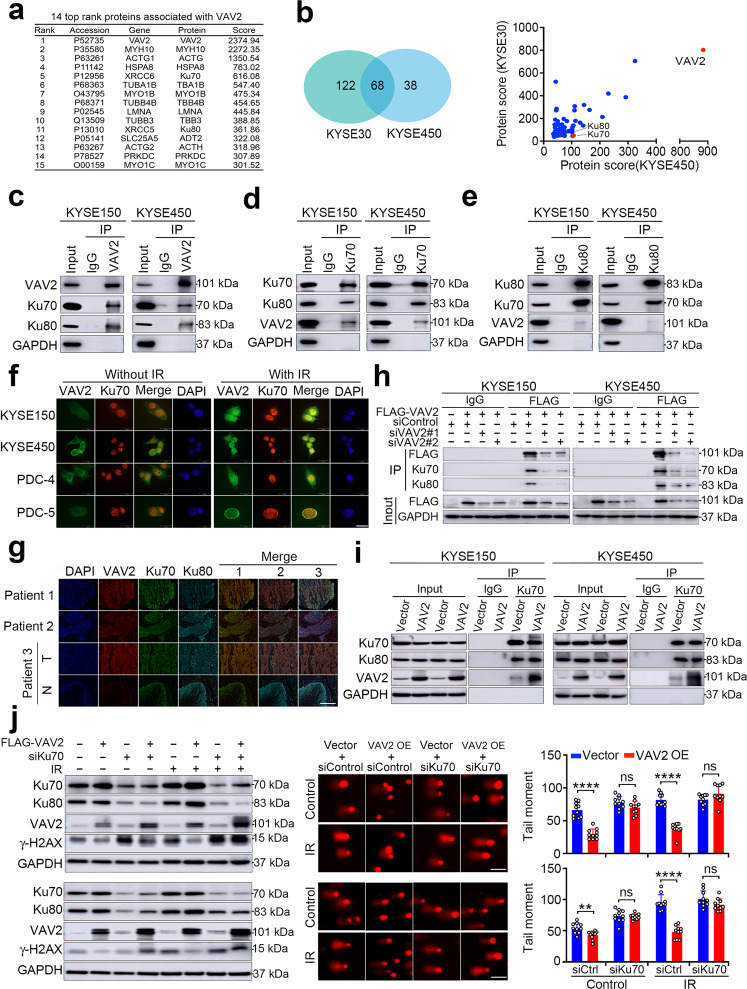
VAV2 is necessary in Ku70 and Ku80-mediated DNA NHEJ repair. **a** Fourteen top proteins potentially associate with VAV2 identified by mass spectrometry in ESCC cells ectopically overexpressing FLAG-tagged VAV2. Cell lysates were immunoprecipitated with antibody against FLAG and antibody against IgG was used as negative control. **b** Proteins potentially associate with VAV2 in KYSE30 and KYSE450 cells. *Left panel* is a Venn diagram showing 68 proteins identified in both cell lines. *Right panel* shows score of each protein. **c**–**e** Western blot detection of VAV2, Ku70 and Ku80 proteins by reciprocal immunoprecipitation with antibody against VAV2 (**c**), Ku70 (**d**) or Ku80 (**e**) in KYSE150 and KYSE450 cells. IgG was used as control. **f** Immunofluorescence analysis of VAV2 and Ku70 co-staining in KYSE150, KYSE450, PDC-4 and PDC-5 cells with or without IR (4 Gy), showing colocalization of VAV2 and Ku70. DAPI was used to label the nucleus. Scale bars, 20 µm. **g** Multiple immunofluorescences analysis of VAV2, Ku70, and Ku80 in clinical ESCC tumor tissues. All tumors (T) show strong colocalization signal of the 3 proteins while the corresponding non-tumor tissues (N) from patient 3, which had low VAV2, show very weak colocalization signal. Merge 1 represents the merge of VAV2 and Ku70, Merge 2 represents the merge of VAV2 and Ku80 and Merge 3 represents the merge of VAV2, Ku70, and Ku80. Scale bars, 100 µm. **h** Western blot analysis of FLAG-VAV2, Ku70 and Ku80 in ESCC cells ectopically expressing FLAG-tagged VAV2 and treated with siVAV2. Cell lysates were immunoprecipitated with antibody against FLAG or IgG. **i** Western blot analysis of VAV2, Ku70, and Ku80 in ESCC cells overexpressing VAV2. Cell lysates were immunoprecipitated with antibody against Ku70 or IgG. **j** Effects of increasing VAV2 expression and decreasing Ku70 expression on DNA damage in ESCC cell lines KYSE150 (*upper panels*) and KYSE450 (*lower panels*). *Left panels* show Ku70, Ku80, VAV2 and γ-H2AX levels detected by Western blot analysis in cells with different treatment. *Middle panels* are image of comet assays showing overexpression of VAV2 significantly diminished DNA damage, which could be reversed by knockdown of Ku70 expression. *Right panel* are the statistics of tail moments in comet assays. OE, overexpression. Data are mean ± SEM from 3 replicate experiments and 10 fields were randomly selected for each experiment. **P < 0.01; ****P < 0.0001 and ns, not significant of Student’s *t*-test

To seek how VAV2 may function in DNA repair, we first performed immunoprecipitation assays using the lysates of KYSE150 cells overexpressing FLAG-VAV2 and anti-FLAG antibody, followed by mass spectrometry analysis. The results indicated that at least 186 proteins might interact with VAV2 (Supplementary Table S4) and among the top 15 ranked by the abundance were X-ray repair cross-complementing protein (XRCC) 6 and 5, also known as Ku70 and Ku80 (Fig. 4a), two proteins function as an obligate Ku70/Ku80 heterodimer in DNA NHEJ repair.^22,23^ The interaction of VAV2 with the Ku70/Ku80 complex were confirmed by the same immunoprecipitation-coupled proteomics assays using anti-VAV2 antibody and cell lysates from natural KYSE30 and KYSE450 cells (Fig. 4b and Supplementary Table S4). We then performed reciprocal co-immunoprecipitation assays with antibody against VAV2, Ku70 or Ku80 and the results further verified the interaction of these three proteins in ESCC cells (Fig. 4c–e). Protein truncation mapping assays showed that it was the SH3 domain in *C*-terminal of VAV2 that mediates the binding between VAV2 and Ku70/Ku80 and it seems that VAV2 binds Ku70 rather than Ku80 since only tiny amount of Ku80 versus Ku70 was coprecipitated with VAV2 (Supplementary Fig. S4a, b), these results are very similar to that for VAV1, another VAV family member containing the same carboxy SH3 domain.^24^ Immunofluorescent staining of ESCC cell lines and primary ESCC cells PDC-4 and PDC-5 showed that VAV2 was distributed in both cytoplasm and nucleus while Ku70 and Ku80 was distributed in the nucleus; however, in the nucleus, VAV2 and Ku70/Ku80 were obviously colocalized. In addition, cells exposed to IR showed more intensive colocalization signal than cells without exposure to IR (Fig. 4f and Supplementary Fig. S4c), suggesting that IR treatment increased VAV2 expression and nuclear translocation, leading to enhanced physiological interaction with Ku70/Ku80 in the nucleus. The stronger interaction of VAV2 with Ku70/Ku80 was also evident in clinical ESCC tumor specimens compared with adjacent normal tissues as shown by multiple immunofluorescent staining (Fig. 4g). In addition, we found that the interaction between VAV2 and Ku70/Ku80 in ESCC cells could be altered by forced *VAV2* expression change: silencing *VAV2* expression by siRNA substantially reduced the interaction (Fig. 4h), but overexpression of *VAV2* substantially enhanced the interaction (Fig. 4i). We then knocked down *Ku70* expression in cells overexpressing *VAV2* to check whether the DNA repair effect is accomplished by the interaction of VAV2 with Ku70/Ku80 and found that depletion of Ku70 significantly suppressed *VAV2* overexpression-caused decrease in γ-H2AX foci and DNA comet tail moments caused by IR (Fig. 4j). Altogether, these results demonstrate that VAV2 participates in Ku70/Ku80-mediated DNA NHEJ repair and its upregulation may enhance the repair ability.

### VAV2 overexpression excessively upregulates STAT1 signaling

**Fig. 5:**
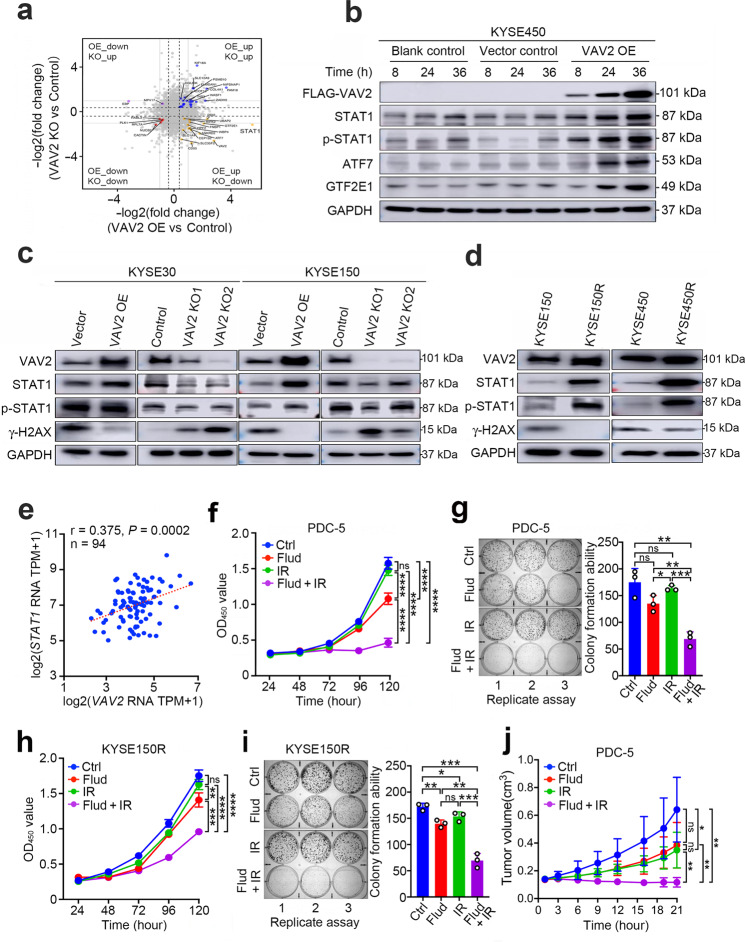
VAV2 overexpression excessively activates STAT1 signaling. **a** Scatter diagram shows the overlapped proteins in KYSE150 cells with VAV2 overexpression (OE) or knockout (KO). Orange, proteins upregulated in cells with VAV2 OE and downregulated in cells with VAV2 KO; Blue, proteins upregulated in cells with VAV2 OE or KO; Purple, proteins downregulated in cells VAV2 OE and upregulated in cells with VAV2 KO and Red, proteins downregulated in cells with VAV2 OE or KO. *P* < 0.05 of Student’s *t*-test compared with that in cells without VAV2 expression change (Control). The level changes of proteins in gray were not statistically significant between treated and control cells (*P* > 0.05). **b** Western blot analysis shows upregulation of STAT1, ATF7 and GTF2E1 in KYSE450 cells with VAV2 OE. p-STAT1, phosph-STAT1. **c** Western blot analysis of total STAT1, phosph-STAT1 (p-STAT1) and γ-H2AX levels in KYSE30 and KYSE150 cells with VAV2 OE or KO. **d** Comparison of VAV2, STAT1, p-STAT1, and γ-H2AX levels in KYSE150, KYSE450, irritation (IR)-induced radioresistant KYSE450R and KYSE450R cells. **e** Spearman correlation of *STAT1* and *VAV2* mRNA levels in clinical ESCC samples. **f**–**i** STAT1 inhibitor Fludarabine (Flud) significantly enhanced the radiosensitivity of radioresistant ESCC cells to IR in vitro. Shown are inhibitory effects of IR (2 Gy), Fludarabine (0.1 and 0.05 μM for cell growth or colony formation assays, respectively) or combination of IR and Fludarabine on cell growth detected by CCK-8 assays (**f**, **h**) and colony formation (**g**, **i**) of PDC-5 and KYSE150R. Data are mean ± SEM from 3 experiments and each had 3 replications. *P < 0.05; **P < 0.01; ***P < 0.001; ****P < 0.0001 and ns, not significant of Student’s *t*-test. **j** STAT1 inhibitor Fludarabine (40 mg/kg) significantly enhanced the radiosensitivity of mouse xenografts derived from radioresistant PDC-5 to IR (10 Gy). Shown are curves of tumor growth overtime and tumors from each mouse in different groups are shown in Supplementary Fig. S5m. Data are mean ± SD from five mice. *P < 0.05; **P < 0.01 and ns, not significant of Student’s *t*-test. See methods for Fludarabine and IR treatment

Although we have elucidated the role of VAV2 in DNA NHEJ repair via cooperating with Ku70/Ku80, the downstream molecular events that might be targeted by potential drugs or inhibitors are not evident and there are currently no inhibitors for VAV2 or Ku70/Ku80 complex. We therefore conducted a proteomics screening in *VAV2* expression changed (overexpression or knockout) and unchanged control cells to seek the further pathways. Differential expression analysis revealed 101 upregulated proteins (FDR < 0.05, FC > 1.3) but 38 downregulated proteins (FDR < 0.05, FC < 0.7) in *VAV2*-overexpressing cells versus control cells and 775 downregulated proteins (FDR < 0.05, FC < 0.7) but 1,060 upregulated proteins (FDR < 0.05, FC > 1.3) in *VAV2*-knocked out cells versus control cells (Supplementary Fig. S5a–d and Supplementary Table S5). Gene ontology analysis indicated that these differentially expressed proteins were mainly enriched in transcription regulatory region DNA binding and RNA binding (Supplementary Fig. S5e, f). Combined analysis found 15 proteins positively correlated with VAV2 in both *VAV2*-overexpressing and *VAV2*-silencing cells and among them were transcription factor STAT1, ATF7 and GTF2E1 (Fig. 5a), whose time-dependent upregulation in *VAV2*-overexpressing cells were verified by Western blotting (Fig. 5b). Since STAT1 signaling can be induced by DNA DSBs via Ku70/Ku80 complex^25^ and might serve as a new cancer therapeutical target,^26,27^ we thus focused on investigating whether *VAV2* overexpression can stimulate STAT1 signaling. We found that both STAT1 and phaspho-STAT1 levels were increased in cells overexpressing *VAV2* but declined in cells with *VAV2* silenced, which is reversely correlated with γ-H2AX level (Fig. 5c). In addition, the STAT1 levels were positively correlated with the levels of VAV2 in ten ESCC cell lines (*r* = 0.734, *P* = 0.0156; Supplementary Fig. S5g) and were substantially higher in IR-induced radioresistant KYSE150 and KYSE450 cells having high VAV2 expression compared with that in the parental cells having lower VAV2 expression and sensitive to IR (Fig. 5d). We also detected positive correlations between VAV2 and STAT1 RNA levels in our previous study samples (*r* = 0.375, *P* = 0.0002, *n* = 94; Fig. 5e) and TCGA ESCC dataset (*r* = 0.301, *P* = 0.0032, *n* = 94; Supplementary Fig. S5h) and protein levels in ESCC samples (*r* = 0.332, *P* < 0.0001, *n* = 240; Supplementary Fig. S5i).

On the basis of these results, we finally examined whether inhibition of the STAT1 activity may reverse the radioresistance of ESCC cells caused by VAV2 overexpression. We treated radioresistant primary ESCC cells (PDC-4 and PDC-5) and radioresistant ESCC cell lines (KYSE150R and KYSE450R) with IR or STAT1 silence and found that silencing STAT1 expression significantly increased the radiosensitivity (Supplementary Fig. S6). In addition, we also treated these cells with IR, STAT1 inhibitor Fludarabine or combination of IR and Fludarabine and found that IR or Fludarabine along had little effect on inhibiting the cell proliferation; however, combined treatment of IR with a small dose of Fludarabine significantly suppressed cancer cell survival and growth (Fig. 5f–i and Supplementary Fig. S7a, b). Parallelly, we observed almost complete suppression of STAT1 activation by Fludarabine in ESCC cells (Supplementary Fig. S7c). These in vitro results indicate that Fludarabine may assist IR to kill radioresistant ESCC cells overexpressing VAV2, which were confirmed by the assays in vivo in mouse xenografts derived from KYSE150 overexpressing VAV2 (Supplementary Fig. S7d) or radioresistant PDC-5 cells (Fig. 5j and Supplementary Fig. S7e). To verify the role of Ku70/Ku80 complex in upregulating STAT1 by VAV2, we silenced Ku70 expression in cells overexpressing VAV2 and found that the STAT1 expression levels were substantially decreased (Supplementary Fig. S7f). In addition, we found that the level of phosphorylated DNA-PKcs (S2056) protein is substantially increased in ESCC cells with VAV2 overexpression, further support that VAV2 overexpression enhances DNA NHEJ repair (Supplementary Fig. S7f). These results demonstrate that it may be the VAV2-Ku70/Ku80 complex that upregulates STAT1 signaling.

### VAV2 predicts radiotherapy vulnerability of ESCC and other types of cancer

**Fig. 6:**
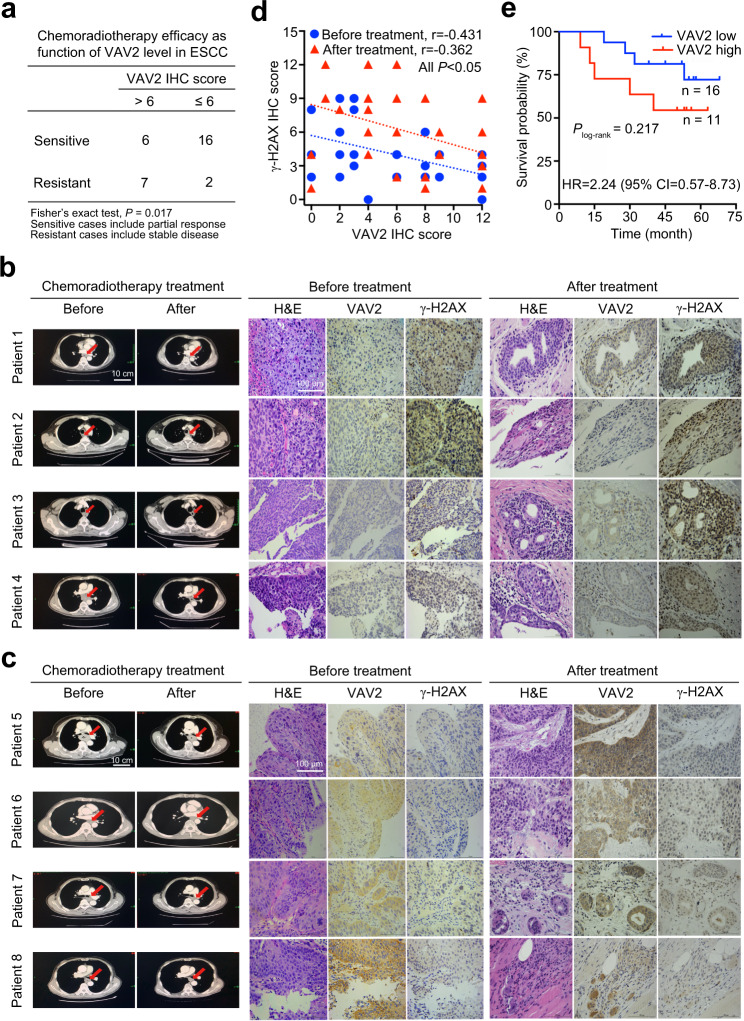
VAV2 predicts radiotherapy vulnerability of ESCC. **a** Chemoradiotherapy efficacy in patients with ESCC (*n* = 31) as function of the VAV2 level in tumor samples detected by immunohistochemical (IHC) staining, showing ESCC having IHC score >6 were significantly more resistant to the therapy compared with ESCC having the score ≤ 6. **b**–**d** Correlation between the VAV2 and γ-H2AX levels in ESCC tumors before and after chemoradiotherapy. Images show computed tomography of tumors before and after treatment (*left*) and H&E, VAV2 and γ-H2AX IHC staining in tumors before (*middle*) and after (*right*) treatment in ESCC sensitive (**b**) or resistant (**c**) to chemoradiotherapy. Scale bars, 100 µm. **d** Shown are the Spearman correlations between VAV2 and γ-H2AX levels in tumors before and after treatment. **e** Kaplan–Meier curves of patient survival according to VAV2 level in ESCC tumor. VAV2 high, IHC score > 6; VAV2 low, IHC score ≤ 6. Also present with the Kaplan–Meier curves is the hazard ratio (HR) and 95% confidence interval (CI) from multivariate Cox proportional hazard models, including age, sex, tumor stage as covariates. Four of 31 patients lost follow-up and thus were excluded in the analysis

We examined VAV2 and γ-H2AX in clinical ESCC samples from 31 patients who received the same concurrent chemoradiotherapy. Among these patients, 9 had partial response (PR) defined as radiosensitive while 22 had stable disease (SD) defined as radioresistant according to the RECIST guidelines for solid tumors.^28^ We found that among the 13 ESCC tumors with VAV2 immunoreaction score (IRS) > 6 (median IRS in all patients), 7 (53.8%) were resistant to chemoradiotherapy; however, among 18 ESCC tumors with VAV2 IRS ≤ 6, only 2 (11.1%) were chemoradiotherapy resistant (Fisher’s exact test, *P* = 0.017; Fig. 6a–c). Moreover, an inverse correlation between VAV2 and γ-H2AX levels in ESCC tumor samples existed (Spearman *r* = −0.431, *P* = 0.015; Fig. 6d and Supplementary Table S6). Based on the above-described results, it is reasonable to believe that high VAV2 expression in ESCC tumor contributed in part to enhanced repair of DNA damages caused by irradiation and therefore conferred tumor to radiotherapy treatment although the effect of chemotherapy drugs was not completely excluded. We also analyzed the correlation between the VAV2 level in ESCC and survival time in this set of patients and found that patients with VAV2 IRS > 6 in ESCC had survival time shorter than those with VAV2 IRS ≤ 6 in ESCC (Fig. 6e), with adjusted hazards ratio (HR) of death being 2.24 (95% CI = 0.57–8.73) although the statistics was not significant (log-rank *P* = 0.217) probably due to relatively small sample size.

By analyzing the TCGA data using gene expression profiling interactive analysis,^29^ we found that *VAV2* is overexpressed in most types of cancer compared with normal tissues (Supplementary Fig. S8a), indicating that VAV2 is a featured oncogene. We investigated the survival association of *VAV2* mRNA levels in TCGA patients who were treated with radiotherapy and had survival data (*n* = 2390 of 20 cancer types) and the results showed that patients (*n* = 1195) having the *VAV2* mRNA level > 1144 (median) in tumor had an HR of 1.20 (95% CI = 1.02–1.41, *P* = 0.028) for death compared with those (*n* = 1195) having the *VAV2* mRNA level ≤ 1144, adjusted for age, sex, tumor stage, and tumor type (Supplementary Table S7). In addition, we also looked at the studies registered in the Gene Expression Omnibus (GEO) database having the information of tumor response to radiotherapy and found a study of rectal cancer (GSE119409) showing that radio-nonresponsive tumors had significantly higher *VAV2* RNA levels than radio-responsive tumors (Supplementary Fig. S8b). Altogether, these results indicate that high VAV2 expression in tumor is closely linked to low efficacy of radiotherapy and poor survival of patients and can be considered as a cancer radiotherapy response marker.

## Discussion

**Fig. 7:**
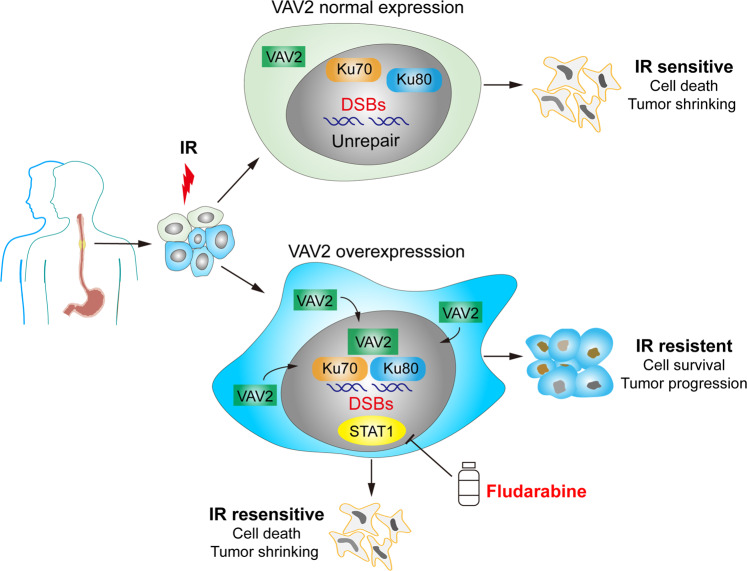
The schematic illustration for the possible mechanisms of VAV2-mediated radioresistance of ESCC cells and Fludarabine as a radiosensitizer. Overexpressed VAV2 in cancer cells increases VAV2-Ku70/Ku80 complex formation and activates signal transducer and activator of transcription 1 (STAT1) signaling, which enhances the ability of cells to repair DNA damage caused by ionizing radiation (IR) and thus promotes cancer growth. However, Fludarabine can inhibit STAT1 activity and reverses the vulnerability of VAV2-overexpressing cancer cells to IR. On the other hand, cancer cells without VAV2 overexpression are relatively sensitive to IR

Radiotherapy is a main treatment option for many types of cancer including locally advanced or unresectable ESCC; however, primary or secondary radioresistance often occur and is the principal cause of cancer relapse and poor prognosis.^14^ Currently, the underlying mechanism for radioresistance is still not well understood, which restricts the efficacy of radiotherapy treatment. In the present study, taken ESCC as a study model, we have identified VAV2 as a key player for radioresistance via integrative analysis of genes associated with radioresistance in mouse PDX models and genes overexpressed in tumor. We have found that high expression of VAV2 significantly impedes tumor response to radiotherapy and is associated with poor survival in patients who were treated with radiotherapy. Mechanistically, VAV2 reduces DNA DSBs, as reflected by the cellular γ-H2AX level, probably through facilitating the formation of Ku70/Ku80 complex to enhance the repair of IR-induced DNA damages. In addition, we have found that in VAV2-overexpressed tumors, STAT1 level is aberrantly upregulated and is also involved in radioresistance. Inhibition of STAT1 signaling by Fludarabine significantly promotes the vulnerability of ESCC cells to IR in vitro and in vivo in PDX models. Hence, the VAV2-STAT1 pathway may serve as a target for improving the efficacy of radiotherapy (Fig. 7).

In this study, we have identified for the first time that VAV2 is required for DNA NHEJ repair and, thus, its overexpression is implicated in resistance of tumor to radiotherapy. Previous studies have reported that VAV2 may promote cancer cell proliferation and invasion in several types of human cancer.^30–34^ Our results are consistent with these findings showing that high VAV2 level in ESCC is significantly correlated with tumor progression and poor survival in patients. However, why and how VAV2 plays a role in clinical outcomes of cancer such as ESCC is unclear yet. Most of the previous studies have mainly focused on the function of VAV2 as a guanine nucleotide exchange factor and suggested that it promotes tumor proliferation and invasion probably through the RAC1, CDC42 and RhoA pathways.^31,35,36^ However, in the present study, we have clearly demonstrated that VAV2 also participates in DNA NHEJ repair. In view of this newly identified VAV2 function, one may expect that tumors having VAV2 overexpression are resistant to radiotherapy. By IHC staining analysis, we found that about half of ESCC samples in the present study had VAV2 overexpression, suggesting that this genomic event may be attributable to a significant proportion of ESCC that are primarily resistant to radiotherapy. This notion has been confirmed by the analysis of our clinical patients who were treated with radiotherapy (Fig. 6a–c). Recurrent VAV2 overexpression may reflect the cellular response to the oncogenic genome alterations in cancer cells. It has been shown that genomic structural alterations such as chromothripsis, a pervasive event across cancers,^6,7^ can induce DNA NHEJ repair,^8^ which may mechanistically require VAV2 upregulation. VAV2 overexpression and its function in DNA repair have also been observed in our radioresistant cell models established by repetitive exposures to IR known to induce DNA DSBs. These results not only provide additional evidence supporting the role of VAV2 in NHEJ repair resulted from DNA DSBs, but also imply that VAV2 overexpression may be a critical mechanism for IR-induced secondary radioresistance. Therefore, VAV2 expression level might serve as a biomarker for predicting radiosensitivity of ESCC and perhaps some other types of cancer in view of the results we have obtained from the pan-cancer analysis.

Mechanistically, we have demonstrated that VAV2 participates in DNA double-strand break repair by physically interacting with Ku70/Ku80 to form a protein complex that is essential for starting NHEJ repair.^22,23^ We have revealed that VAV2 overexpression and nuclear translocation substantially enhances the binding of Ku70 and Ku80 and significantly reduces IR-induced DNA damages while the expression levels of both Ku70 and Ku80 themselves are not affected by VAV2. On the other hand, when Ku70 expression is knocked down, IR-caused DNA damages are increased in cells even VAV2 is overexpressed. These results suggest that VAV2 might not have the activity directly performing DNA repair; instead, it might serve as other functions to facilitate the formation of Ku70/Ku80 complex. Based on our results, we propose that VAV2 might function as an adaptor protein for binding of Ku70 to Ku80 although this notion needs further validation. However, how VAV2 translocates into the nucleus in response to IR is not currently evident and needs more efforts to dissect. The interactions of Ku70/Ku80 with other proteins have been documented. For example, Ku70 has been shown to be bound by VAV1, another VAV family protein that has the similar C-terminal SH3 domain,^24^ although the biological relevance of their binding has not been characterized yet. Several other studies have reported that Ku70/Ku80 may physically interact with some proteins, resulting in enhanced DNA double-strand break repair.^37–41^ These findings strongly support our results in the present study. We have also found that the level of the activated DNA-PKcs, another important protein involved in NHEJ repair, is substantially increased in ESCC cell lines with VAV2 overexpression, further confirming that VAV2 overexpression enhances DNA NHEJ repair. However, it worth noting that a recent study has shown that LRRC31, a tumor suppressor protein, can directly bind Ku70/Ku80 to inhibit DNA repair and sensitizes breast cancer brain metastasis to radiotherapy.^42^ Furthermore, we have observed that upon VAV2 expression alternation in ESCC cells, the expression pattern of some genes in MYC target, E2F target, apoptosis, and DNA repair pathways including homologous recombination (HR) pathway also changed, which might at least in part be attributable to the STAT1 expression or activation change. It would be interesting to clarify this issue in the future investigations.

Another interesting result is that by using proteomics analysis, we have found that STAT1 was significantly upregulated in cells overexpressing VAV2. STAT1, the master transcription factor of interferon-related intracellular signaling, has been widely explored in cancer progression and is predominantly considered as a tumor suppressor. However, a bulk of evidence also indicates STAT1 as a tumor promoter in the specific context.^43–47^ It has been reported that DNA DSBs can trigger the activation of STAT1 signaling^25^ and aberrant STAT1 activation may confer protection from IR in cancer cells.^26,45,47^ In the present study, we have observed that STAT1 is substantially upregulated in ESCC cells with high VAV2 but low γ-H2AX (Fig. 5), suggesting that STAT1 may be regulated by VAV2 or VAV2/Ku70/Ku80 complex but not directly associated with the events of DNA DSBs. This result is a novel finding for the development of radioresistance by STAT1 activation, although how VAV2 regulates STAT1 signaling is unclear in the present study and warrants further investigations. Although VAV2 inhibitor is currently unavailable, the STAT1 signaling pathway is druggable and may serve as an alternative target for reversing radioresistance of cancer cells. Indeed, we have demonstrated that treatment of STAT1 inhibitor Fludarabine significantly promoted the sensitivity of radioresistant ESCC cell lines and mouse xenografts generated from patient-derived ESCC cells to radiotherapy. It has been shown that Fludarabine can specifically deplete STAT1 and inhibits STAT1 activation,^48^ which might block the activity of the VAV2-STAT1 axis participated in the DNA NHEJ repair. Furthermore, Fludarabine is a nucleotide analog that can be phosphorylated to form Fludarabine triphosphate that competes with natural nucleotides and incorporate into DNA strand. Fludarabine can also inhibit ribonucleotide reductase activity and thus diminish the dATP pool and DNA synthesis.^49,50^ In light of these findings, one may expect that Fludarabine could be a potent sensitizer for radiotherapy of cancer with VAV2 overexpression. This novel finding may persuade clinical trials since it is an FDA approved chemotherapeutic drug used in the treatment of chronic lymphocytic leukemia.^51^

In summary, through multi-omics analysis and functional assays, we have identified VAV2 that plays a critical role in radioresistance. We have provided a new insight into the molecular mechanism for VAV2 actions in radioresistance that is to mediate Ku70/Ku80 complex formation to facilitate NHEJ repair of IR-induced DNA damages and activate STAT1 signaling perhaps to help more complicated cellular DNA repair processes. We have also revealed that the VAV2-STAT1 axis can be targeted by STAT1 inhibitor to enhance radiosensitivity of cancer overexpressing VAV2, which might have an important implication in developing more effective and precision cancer radiotherapy.

## Materials and methods

### Study subjects and tissue specimens

Fresh ESCC tumor specimens for preparing PDXs (obtained in 2020) and formaldehyde-fixed and paraffin-embedded tissues samples for making tissue arrays (obtained between 2015 and 2016) were collected from patients underwent surgery in Linzhou Cancer Hospital, Henan Province, China. All patients did not receive chemotherapy or radiotherapy before surgery and ESCC were histopathologically confirmed. The relevant characteristics and clinical information were obtained from medical records (Supplementary Table S2) and most part of this tissue array samples has been published previously.^16^ Written informed consents were obtained from all individuals and this study was approved by Chinese Academy of Medical Sciences Cancer Hospital. We also recruited 31 patients with ESCC received the same concurrent chemoradiotherapy regimen, i.e., intensity-modulated radiation therapy (40−50 Gy) and paclitaxel combined with cisplatin or nedaplatin before surgery at Chinese Academy of Medical Sciences Cancer Hospital, Beijing, China. The response of tumor to concurrent chemoradiotherapy was evaluated by computed tomography according to the RECIST guidelines for solid tumors:^28^ complete response (CR) and partial response (PR) were defined as sensitive while stable disease (SD) and progressive disease (PD) were defined as resistant (Supplementary Table S6). Tumor specimens were obtained by surgery or biopsy before chemoradiotherapy. Patient survival time was measured from the date of end of chemoradiotherapy to the date of last follow-up or death. Whether and when a patient had died was obtained from inpatient records, patient’s family or through follow-up call. Patients were followed up for a maximum of 68 months and during this period, 4 patients lost follow-up. Thus, only 27 patients were included for the survival analysis. Written informed consent was obtained from all individuals and this study was approved by the Chinese Academy of Medical Sciences Cancer Hospital.

### Cell lines and reagents

Cell lines KYSE30, KYSE150, KYSE450 were kind gifts from Dr. Y. Shimada of Hyogo College of Medicine, Japan. They were authenticated by DNA finger printing analysis and tested free of mycoplasma infection. Cells were cultured in RPMI 1640 medium supplemented with 10% fetal bovine serum (FBS) at 37 °C in a humidified incubator with 5% CO_2_. The STAT1 inhibitor Fludarabine obtained from Selleck (S1491) was dissolved in dimethylsulfoxide (DMSO) at an appropriate concentration as a stock solution and stored at −80 °C before further use.

### Plasmids and small-interfering RNA transduction

FLAG-tagged *VAV2* expression plasmid (#58833-1) was from Genechem. Small-interfering RNAs (siRNAs) targeting *VAV2* (#1, ucacagaggccaagaaauu; #2, gaaagucugccacgauaaa and #3, gggacgacaucuacgagga) and *Ku70* (#1, gagugaagaugaguugacatt; #2, gguuaaagcugaagcucaatt and #3, gacauauccuuguucuacatt) were from ViewSolid Biotech. The control sequence was uucuccgaacgugucacgutt. The transfection of plasmid and siRNAs was performed using Lipofectamine 2000 (Invitrogen). The RNA interfering-based high-content screening assays were performed as described previously^16^ with siRNAs shown in Supplementary Table S8.

### Establishment of PDX in mice and radiotherapy

Surgically removed fresh human ESCC tumor sample (F0 tumor) was implanted subcutaneously in NOD/SCID/IL-2Rγnull (NSG) mice (Beijing IDMO Co.) for tumor expansion (F1 tumor). If necessary, F2 tumor was made in another mouse using F1 tumor. When tumor expanded to a size of ~800 mm^3^, the F1 or F2 tumor was removed, cut into small pieces and implanted again in a group of 5 or 6 mice. Tumor volume was calculated as (L × W^2^)/2, where L and W are the long and short diameters of tumor, respectively. When the xenograft was 200–250 mm^3^, mice were randomly divided into groups as indicated in the corresponding figures and X-irradiated (3.5 Gy/min) once every other day for 4 times at 6 Gy each time using a MultiRad225 (Faxitron). Tumor size was measured at least every 3 days and mice were sacrificed when tumor in control group reached 2000 mm^3^. The sensitivity of PDXs to radiotherapy was defined by relative tumor volume (RTV), where RTV = *V*_after IR_/*V*_before IR_. RTV < 1 was defined as radiosensitive and RTV > 1 as radioresistant.^17^ Animal experiments were carried out in compliance with approved protocols and guidelines from the Institutional Animal Care and Use Committee of the Chinese Academy of Medical Sciences.

### Establishment of PDX-derived primary ESCC cells

PDX-derived primary ESCC cells were prepared as described previously.^52,53^ Briefly, PDX was dissected from mouse and gently minced to small fragments at about 2 mm in diameter. Tissue sample was digested in RPMI-1640 medium (Invitrogen) containing collagenase IV (Gibco) and hyaluronidase (Sigma-Aldrich) for 30 min at 37 °C. Cell suspension was filtered through a 70-μm cell strainer, briefly centrifuged for 5 min and the resultant cell pellet was resuspended in advanced DMEM/F12 (Gibco, 12634010) supplemented with B-27 (17504-044), HEPES (15630080), GlutaMAX™ (35050061), epithelial growth factor (PMG8043), 10 µM Rho-associated coiled-coil-containing kinase inhibitor Y-27632 (Selleck Chemicals, S1049) and 5 µM TGFβ inhibitor A83-01 (Tocris Bioscience, 2939). The cells were kept in a humidified 5% CO_2_ incubator at 37 °C.

### Establishment of ESCC xenografts from KYSE150 or PDC-5 in mice and irradiation treatment

Five million KYSE150 cells overexpressing VAV2 or PDC-5 were harvested in 150 µL of PBS containing 50 µL Matrigel and subcutaneously injected into the armpits of 4-week-old female NSG mice. When expanded to a size of ~800 mm^3^, tumor was cut into equally small portions and implanted into the hind legs of mice. When the xenograft reached about 150 mm^3^, mice were randomly divided into groups and treated with irradiation, Fludarabine or irradiation plus Fludarabine. Irradiation (10 Gy) was given on the first day and Fludarabine (40 mg/kg) was concurrently given by intraperitoneal injection for 3 consecutive days. Tumor was measured every week for its volume defined by length x width^2^ x 0.5 and collected when reached to about 2,000 mm^3^ or to the end of follow-up time (21 days).

### Establishment of cell lines with *VAV2* overexpression or knockout

Lentivirus for stable *VAV2* overexpression (#40898-1) was purchased from Genechem as viral particles. KYSE30, KYSE150 and KYSE450 cells were infected with the virus and cultured in complete medium for 24 h followed by selection with puromycin. The CRISPR/Cas9 system was used to generate genomic deletion of *VAV2* in ESCC cell lines. Single-guide RNA (sgRNA) sequences targeting the genomic sequence of *VAV2* designed using the CRISPR design tool were cloned into the pUC19-U6-sgRNA plasmid. The pCAG-Cas9-EGFP and pUC19-U6-sgRNA plasmids were co-transfected into KYSE30, KYSE150 and KYSE450 cells, and the fluorescent cells were sorted by flow cytometry into a 96-well plate for culture. The efficiency of *VAV2* overexpression or knockout was examined by Western blotting assays.

### RNA-sequencing analysis

Total RNA in cell lines or tissue samples was extracted using TRIzol and subjected to sequencing. RNA-sequencing data were mapped to the GRCh38 human genome by HISAT2 (version 2.1.0). The gene expression matrix of raw read counts after annotation by HTSeq (version 0.6.1p1) was processed using DESeq2 (version 1.22.2). The expression profiles were normalized by the Transcripts Per Kilobase of exon model per Million mapped reads (TPM) method and log2 transformed. Gene set enrichment analysis (GSEA) was performed on normalized RNA-sequencing data using the GSEA software.

### Proteomics analysis

Total cellular protein was extracted for 4D label-free quantification (4D-LFQ) proteomics and Western blotting according to the manufacturer’s instructions. 4D-LFQ proteomics analysis was performed by PTM BIO. Differential expression analysis was used to explore the potential upregulated or downregulated proteins between cells with different treatment.

### Cell viability and colony formation assays

Cell viability in 96-well plates with different treatment was measured using the Cell Counting kit-8 (Dojindo Lab). Colony formation ability in complete growth medium after different treatment was determined by using ImageJ software to count the number of cells, which were fixed with methanol and stained with 0.5% crystal violet. Survival fraction of cells was the ratio of the plating efficiency of treated cells to that of control cells.

### Extreme limiting dilution assays

An extreme limiting dilution assay was performed as described previously.^54^ Different cell dilutions (0, 6.25, 12.5, 25, 50, 100, and 200 per well for KYSE150 and KYSE450 and 0, 12.5, 25, 50, 100, 200, and 400 per well for PDCs) with or without irradiation (2 or 4 Gy) were seeded in 96-well plates. After 7–14 days, the sphere number was counted and analyzed using an online software (http://bioinf.wehi.edu.au/software/elda/).

### Three-dimensional tumor sphere culture

The Matrigel matrix was diluted to 5 mg/mL on ice in precooled primary cell culture medium. PDCs were digested with trypsin into a single-cell suspension and centrifuged at 1000 × *g* for 5 min. Cells were the resuspended in complete medium and adjusted to be of 1–5 × 10^4^ cells/mL. Cell suspension (50 μL) was added to 150 µL of Matrigel matrix (5 mg/mL) and plated in 6-well plates. After incubation at 37 °C for 30 to 45 min, 2 mL of primary cell culture medium was added to each well. Tumor spheres were incubated for 7 to 10 days at 37 °C in a 5% CO_2_ humidified incubator and the 3D tumor spheres were imaged under confocal microscopy.

### Quantitative real-time PCR

Total RNA was extracted from cells using the RNA-Quick Purification Kit (ES Science, RN001). A PrimeScript RT reagent kit and SYBR Premix Ex Taq II kit (Takara) were used for the detection of mRNA expression through an ABI 7900HT Real-Time PCR system using the primers shown in Supplementary Table S9. Individual RNA levels were determined relative to *GAPDH* RNA level.

### Immunohistochemical analysis

Paraffin-embedded tissue sections and tissue arrays were incubated with antibody against VAV2 (1:50), STAT1 (1:5000), γ-H2AX (1:200) or Ki-67 (1:100) at 4 °C overnight and then detected with the ABC Kit (Pierce). The score of labeling intensity was estimated as negative (0), weak (1), moderate (2) or strong (3). The extent of staining, which was defined as the percentage of positively stained cells, was scored as 1 (≤10%), 2 (11−50%), 3 (51−80%) and 4 (>80%). The total immunoreactive score (IRS) was obtained by multiplying the staining intensity score and the staining extent score and ranked from 0 to 12.

### Multiple immunofluorescence assays

Opal multiplex staining was performed according to the Opal 5-Color Manual IHC Kit (PerkinElmer). Opal DAPI, Opal 520, Opal 570, and Opal 650 from Abcam were used to generate different signals corresponding to Ku70, VAV2, or Ku80 antibody, respectively. The slides were counterstained with DAPI for nuclear visualization and subsequently coverslipped using VectaShield Hardset mounting media. The slides were imaged using a Vectra Polaris Automated Quantitative Pathology Imaging System (PerkinElmer). We used inForm software (PerkinElmer) to unmix and remove autofluorescence and to analyze the multispectral images.

### Immunofluorescent staining of γH2AX, VAV2, Ku70, and Ku80

Seeded on coverslips to 70–80% confluency, ESCC cell lines and PDCs were allowed to attach for 12 h and then treated with irradiation generated by an X-ray irradiator MultiRad 225 (Faxitron). At the time points as indicated in corresponding figures, cells were washed with PBS and fixed in 4% paraformaldehyde for 8 min. After three washings, cells were permeabilized with 0.2% Triton X-100. The coverslips were then blocked with 1% BSA in PBS containing 0.1% Triton X-100 and stood for 30 min at room temperature. The blocked coverslips were then probed with antibody against VAV2, Ku70, Ku80 or γ-H2AX, followed by CoraLite594-conjugated secondary antibody (Proteintech, SA00013-3/4). Coverslips were mounted with DAPI (ZSGB-BIO, ZLI-9557). Images of fluorescent γ-H2AX foci, VAV2, Ku70, and Ku80 were captured by confocal microscopy.

### Characterization of binding of VAV2 to Ku70 or Ku80

We designed various truncated proteins of VAV2 based on the functional domains, which was tagged by FLAG and cloned into pcDNA3.1 to yield pcDNA3.1-FLAG-VAV2 for transient overexpression of VAV2. The plasmids were synthesized by TSINGKE. The transient overexpressing plasmids HIS-XRCC6 (#66490-2, producing Ku70) and MYC-XRCC5 (#66491-1, producing Ku80) were purchased from GENECHEM. KYSE150 cells were co-transfected with HIS-XRCC6 or MYC-XRCC5 and various truncations of FLAG VAV2. Cell lysates were subjected to immunoprecipitation and western blotting analysis.

### Immunoprecipitation assays

Cells were lysed in RIPA lysis buffer (Beyotime, P0013D) with PMSF and phosphatase inhibitor cocktail for 30 min at 4 °C. The supernatant was collected by centrifugation at 12,000 × *g* for 15 min. Assay was conducted with Dynabeads^TM^ Protein G Immunoprecipitation Kit (Invitrogen, 10007D) and the resultant was eluted using elution buffer and subjected to SDS-PAGE, Western blotting or MS analysis (PTM BIO). The antibody for co-immunoprecipitation of VAV2 (sc-271442) and Ku70 (sc-17789) were from Santa Cruz while the antibody against FLAG (F1804) or Ku80 (MA5-12933) was from Sigma-Aldrich or Thermo Fisher. Mouse IgG (B900620) and secondary antibody HRP-mouse anti-rabbit IgG (SA00001-7L) were from Proteintech.

### Western blot analysis

Cells were lysed with RIPA lysis buffer (Solarbio, R0020) containing PMSF (Solarbio, P0100), phosphatase inhibitor cocktail I (MCE, HY-K0021) and phosphatase inhibitor cocktail II (MCE, HY-K0022). The protein concentration of each sample was determined by BCA assay using the BCA kit (Thermo Fisher Scientific). Lysate containing 10–20 µg of protein was separated on SDS-PAGE and transferred to PVDF membranes (Millipore). Antibody against VAV2 (ab52640), Ku70 (ab92450), STAT1 (ab234400), phospho-STAT1 (S727) (ab109461), ATF7 (ab183507), GTF2E1 (ab140634), GAPDH (ab181602), HIS (ab213204), DNA PKcs (ab32566) or DNA PKcs (phosphor S2056) (ab124918) was from Abcam while antibodies against γ-H2AX (Ser139) (#2577), Ku80 (#2180), FLAG (#14793) or MYC (#2278) antibody was from Cell Signaling Technology. The signal was detected with a SuperSignal^TM^ West Pico/Femto Chemiluminescent Substrate kit (Thermo Fisher, 34580) through the Amersham Imager 600. The protein bands were quantified by gray scanning using ImageJ software.

### Single-cell gel electrophoresis assays

Single-cell gel electrophoresis (comet assay) was performed to examined DNA DSBs. Briefly, cells were collected and suspended in ice-cold PBS at 2 h which were treated without or with irradiation (4 Gy). A total of 1–2 × 10^4^ cells were prepared for each assay using the Comet Assay DNA Damage Detection Kit (KeyGEN BioTECH). The sample was then analyzed by microscopy using a Cytation5 (BioTek) and tail moment was analyzed using the Gene5 software.

### Analysis of public data

Data from The Cancer Genome Atlas (TCGA) (http://gdac.broadinstitute.org) were used to analyze the correlations between the *VAV2* mRNA levels and *STAT1* mRNA levels in esophageal cancer and the *VAV2* mRNA levels in different types of cancer and the correlation with patient survival. The Gene Expression Omnibus (GEO) (https://www.ncbi.nlm.nih.gov/geo/query/acc.cgi?) was used to analyze the relationship between the *VAV2* levels and the sensitivity of cancer to radiotherapy.

### Statistical analysis

We used Fisher’s exact test for any independence test between two categorical variables and Wilcoxon rank-sum test for any independence test between a continuous variable and a binary categorical variable, when there was no covariate to adjust for. Otherwise, we used an *F*-test to compare two generalized linear models, one of which included the variable being evaluated as a predictor. Spearman’s rank correlation coefficient was used to measure the correlation between two continuous variables. The results for functional analysis are presented as mean ± SEM and the difference between two groups was examined by Student’s *t*-test. We used the log-rank test in univariate survival analyses and the Cox proportional hazards model in multivariate survival analyses. The Kaplan–Meier plot was used for presentation. All statistical tests were two-tailed and *P* < 0.05 was considered significant.

## Supplementary information

Supplementary_Materials

Supplementary Table 1

Supplementary Table 2

Supplementary Table 3

Supplementary Table 4

Supplementary Table 5

Supplementary Table 6

Supplementary Table 7

Supplementary Table 8

Supplementary Table 9

## Data Availability

All data are available within the article, supplementary information or available from the corresponding author upon reasonable request.
