# Supplementary material for: VAV2 is required for DNA repair and implicated in cancer radiotherapy resistance
Source: Signal Transduct Target Ther. 2021 Aug 30;6:322. doi: 10.1038/s41392-021-00735-9 (PMC8405816; doi:10.1038/s41392-021-00735-9)
Supplement: Supplementary file 1 — Supplementary_Materials [file 41392_2021_735_MOESM1_ESM.docx]

**Supplementary Materials for**

VAV2 is required for DNA repair and implicated in cancer radiotherapy resistance

Weiling Liu^1,8^, Chuanwang Miao^1,8^, Shaosen Zhang^1,8^, Yachen Liu^1^, Xiangjie Niu^1^, Yiyi Xi^1^, Wenjia Guo^2,3^, Jiahui Chu^4^, Ai Lin^1^, Hongjin Liu^1^, Xinyu Yang^1^, Xinjie Chen^1^, Ce Zhong^1^, Yuling Ma^1^, Yuqian Wang^1^, Shihao Zhu^1^, Shuning Liu^1^, Wen Tan^1^, Dongxin Lin^1,5,6,7,^*, Chen Wu^1,6,7,^*

Correspondence to: Chen Wu (chenwu@cicams.ac.cn); Dongxin Lin (lindx@cicams.ac.cn)

**This PDF file includes:**

Supplementary Figures S1 to S8

Captions for supplementary Tables S1 to S9


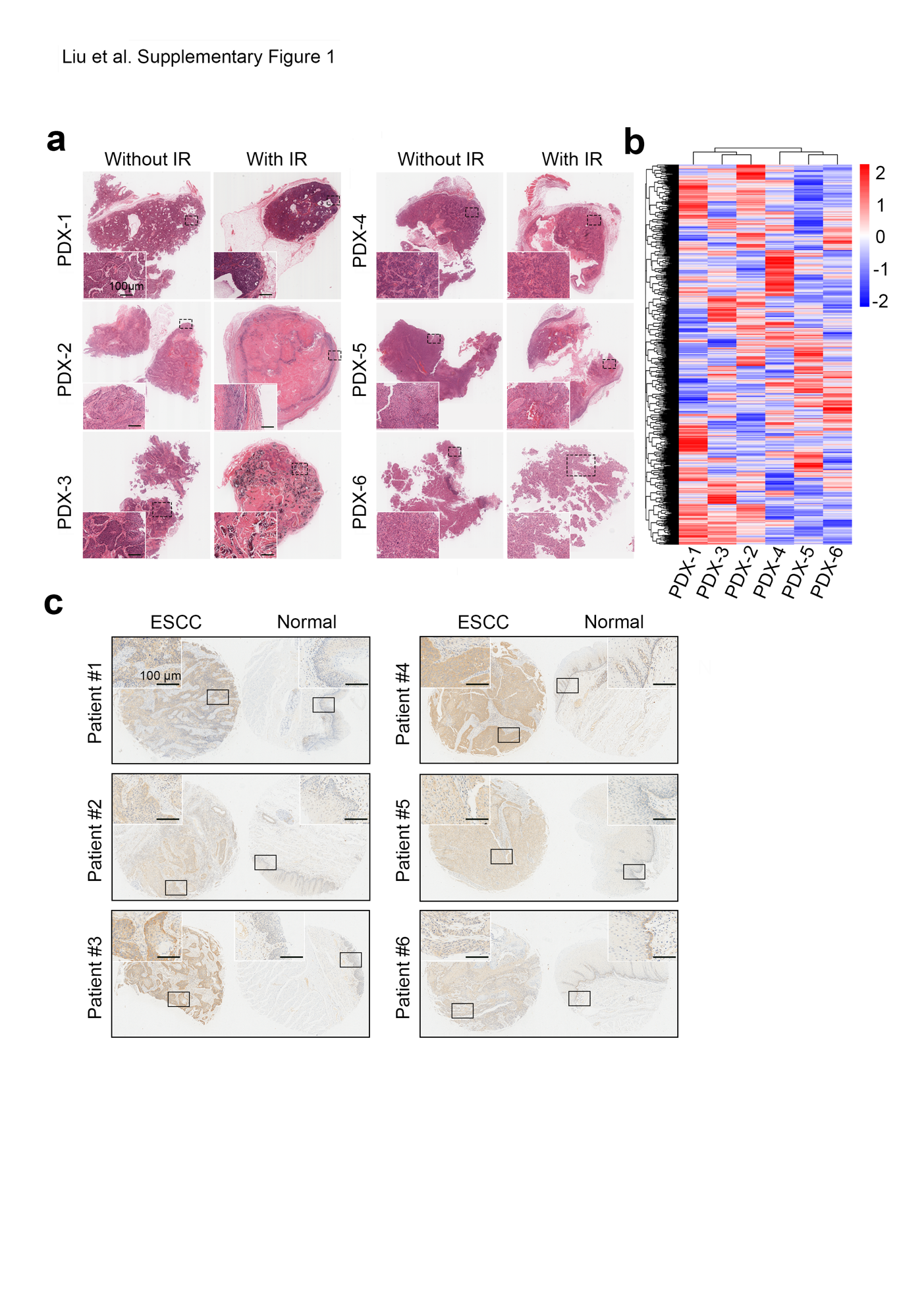


Figure S1. Additional Figures related to Figure 1.

**a** H&E staining of the entire tissue cross-sections of 6 PDXs sensitive or resistant to irradiation (IR). **b** Heatmap of the genome-wide expression matrix cluster analysis of 26,661 genes in the 6 PDXs. **c** Representative images of tissue arrays for immunohistochemical staining of VAV2. Scale bar, 100 μm.


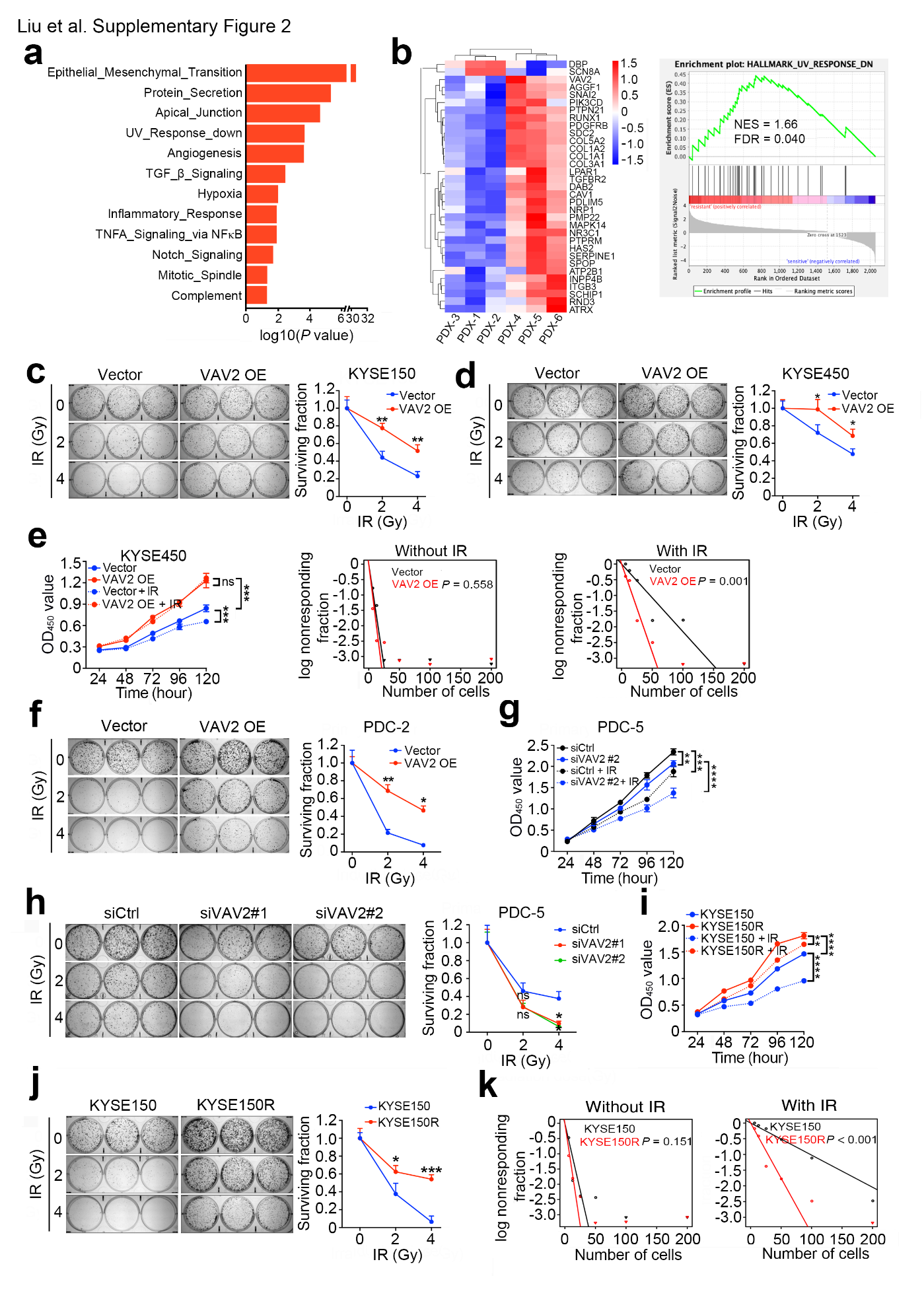


Figure S2. Additional Figures related to Figure 2.

**a** Gene set enrichment analysis showed 12 top significant pathways in radioresistant PDXs identified by RNA-sequencing. **b** Heatmap (*left*) and enrichment plot (*right*) for ultraviolet (UV) response pathway. **c**, **d** Colony formation ability of KYSE150 (**c**) and KYSE450 (**d**) cells with or without *VAV2* overexpression (OE) treated with (2 and 4 Gy) or without irradiation (IR). *Left* *panel* are representative images of colony formation assays while *right panels* are statistics of surviving fraction of treated cells to control cells. **e**, **f** Forced *VAV2* OE in KYSE450 cells (**e**) or radiosensitive PDC-2 cells (**f**) caused resistance of cells to IR (4 Gy). *Left panels* shows proliferation curves of cells and *right panels* shows fractions of cell survival by limiting dilution assays. **g**, **h** Knockdown of *VAV2* expression significantly promoted the sensitivity of PDC-5 cells to IR. **i−k** The differential sensitivity between KYSE150 and radioresistant KYSE150R cells to IR as examined by proliferation curves of cells (**i**), colony formation (**j**) and limiting dilution (**k**) assays. IR dose for proliferation curves of cells and limiting dilution assays were 4 Gy. Data in (**f−j**) are mean ± SEM from at least 3 experiment replications; *, *P* < 0.05; **, *P* < 0.01; ***, *P* < 0.001; ****, *P* < 0.0001 and ns, not significant of Student’s *t*-test.


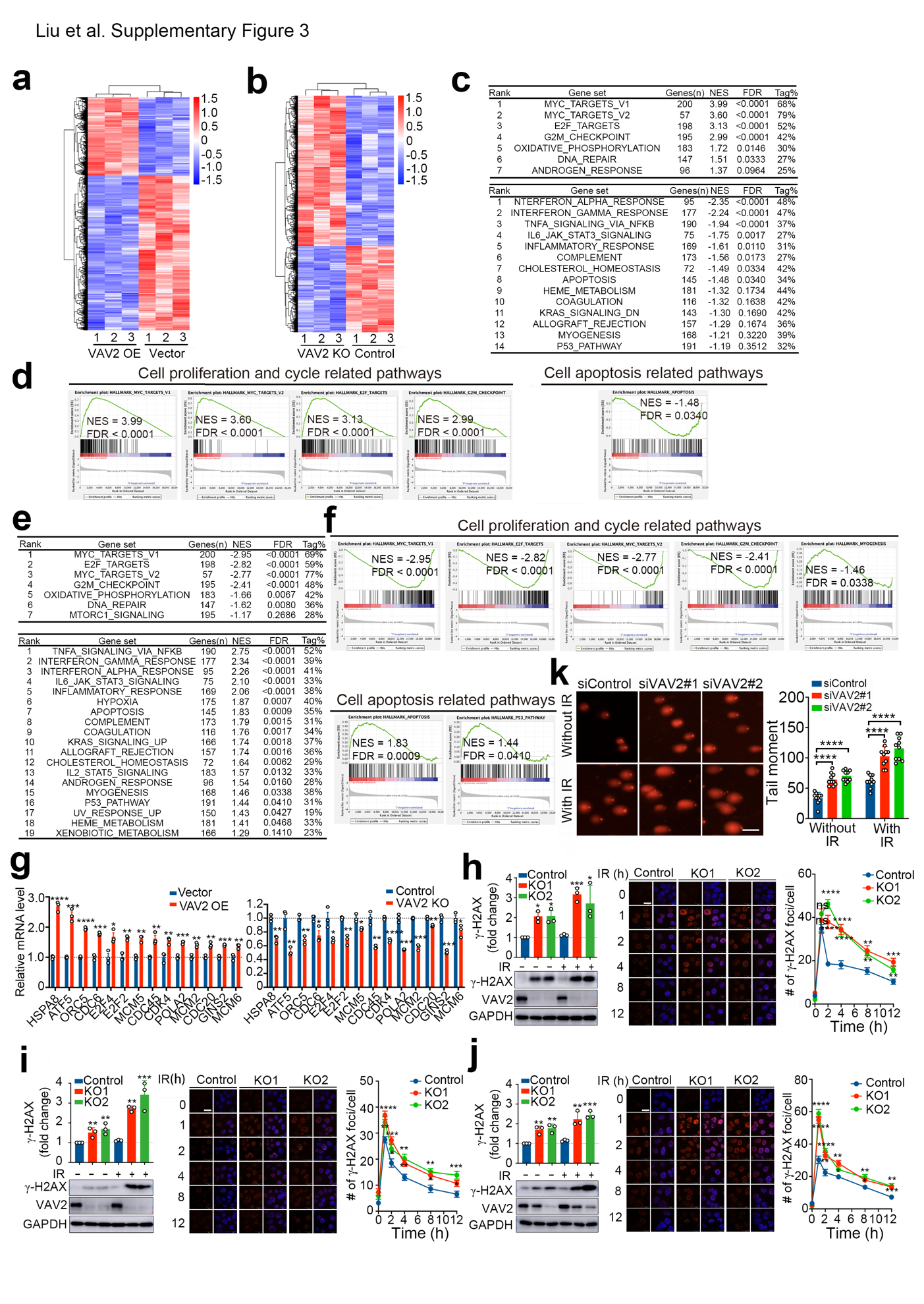
Figure. S3 Additional Figures related to Figure 3.

**a**, **b** Heatmaps of significant expression changes (fold change > 1.3 or < 0.7, *P* < 0.05) of genes in KYSE150 cells with *VAV2* overexpression (OE) or knockout (KO). **c** Significant enrichment of gene sets in KYSE150 cells with *VAV2* OE identified by Gene set enrichment analysis. *Upper,* positively correlated pathways and *lower*, negatively correlated pathways. Tag%, the fraction of genes contributing to the enrichment score. NES, normalized enrichment score. **d** Enrichment plot for the cell proliferation and cell cycle related pathways (*left*) and cell apoptosis related pathways (*right*) in cells with *VAV2* OE. **e** Significant enrichment of gene sets in KYSE150 cells with *VAV2* KO identified by Gene set enrichment analysis. Upper, positively correlated pathways and *lower*, negatively correlated pathways. Tag%, the fraction of genes contributing to the enrichment score. NES, normalized enrichment score. **f** Enrichment plot for the cell proliferation and cell cycle related pathways (*upper*) and cell apoptosis related pathways (*lower*) in cells with VAV2 KO. **g** RT-qPCR analysis of the expression levels of genes in G2M checkpoint pathway in cells with *VAV2* OE *(left*) or KO (*right*). **h**−**j** DNA double-strand breaks expressed by γ-H2AX level in *VAV2*-KO KYSE150 (**h**), KYSE450 (**i**) and KYSE30 (**j**) cells treated with or without irradiation (IR, 4 Gy). *Left panels* show γ-H2AX and VAV2 levels by Western blot analysis in cells 2 hours after IR. *Middle panels* show images of γ-H2AX foci in cells at various time points of IR as indicated. Scale bars, 20 µm. *Right panels* represent the statistics. Data are means ± SEM from 3 replicate experiments. **k** DNA double-strand breaks detected by comet assays in PDC-4 cells with or without VAV2 silenced and treated with or without IR (4 Gy). *Left panel* shows fluorescence images of comet assays. Scale bars, 100 µm. *Right panel* shows the statistics. Data are mean ± SEM from 3 replicates and ten fields were randomly selected for each experiment. *, *P* < 0.05; **, *P* < 0.01; ***, *P* < 0.001 and ****, *P* < 0.0001 of Student’s t-test.


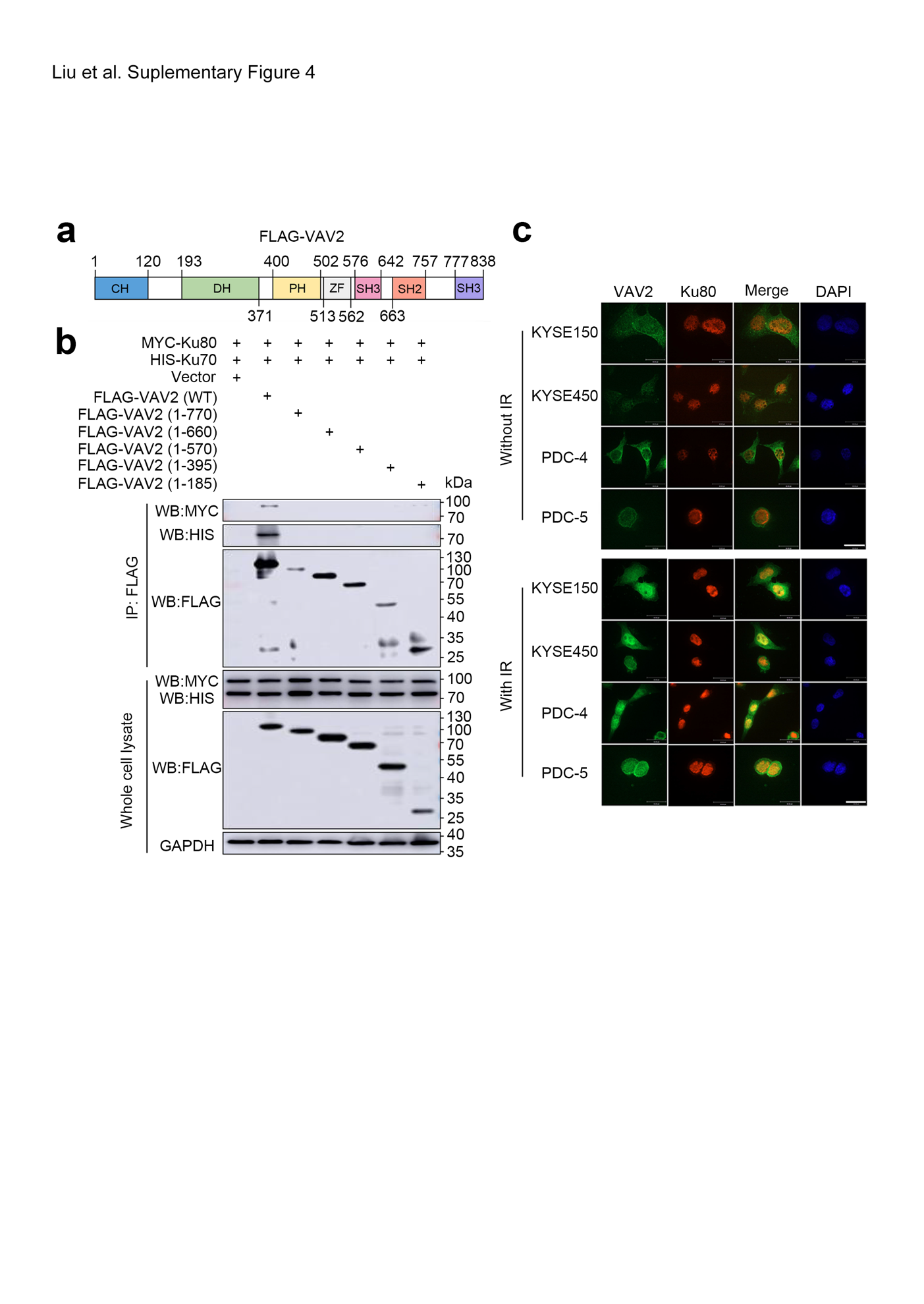


Figure. S4. Additional Figures related to Figure 4.

**a** Schematic diagram of FLAG-tagged VAV2 protein domain structure. **b** Immunoblot analysis of HIS-tagged Ku70 or MYC-tagged Ku80 coimmunoprecipitated with FLAG-tagged full length (WT) VAV2 and its truncated forms. **c** Immunofluorescence analysis of VAV2 and Ku80 co-staining in KYSE150, KYSE450, PDC-4 and PDC-5 cells with or without IR (4 Gy), showing colocalization of VAV2 and Ku80. DAPI was used to label the nucleus. Scale bars, 20 µm.


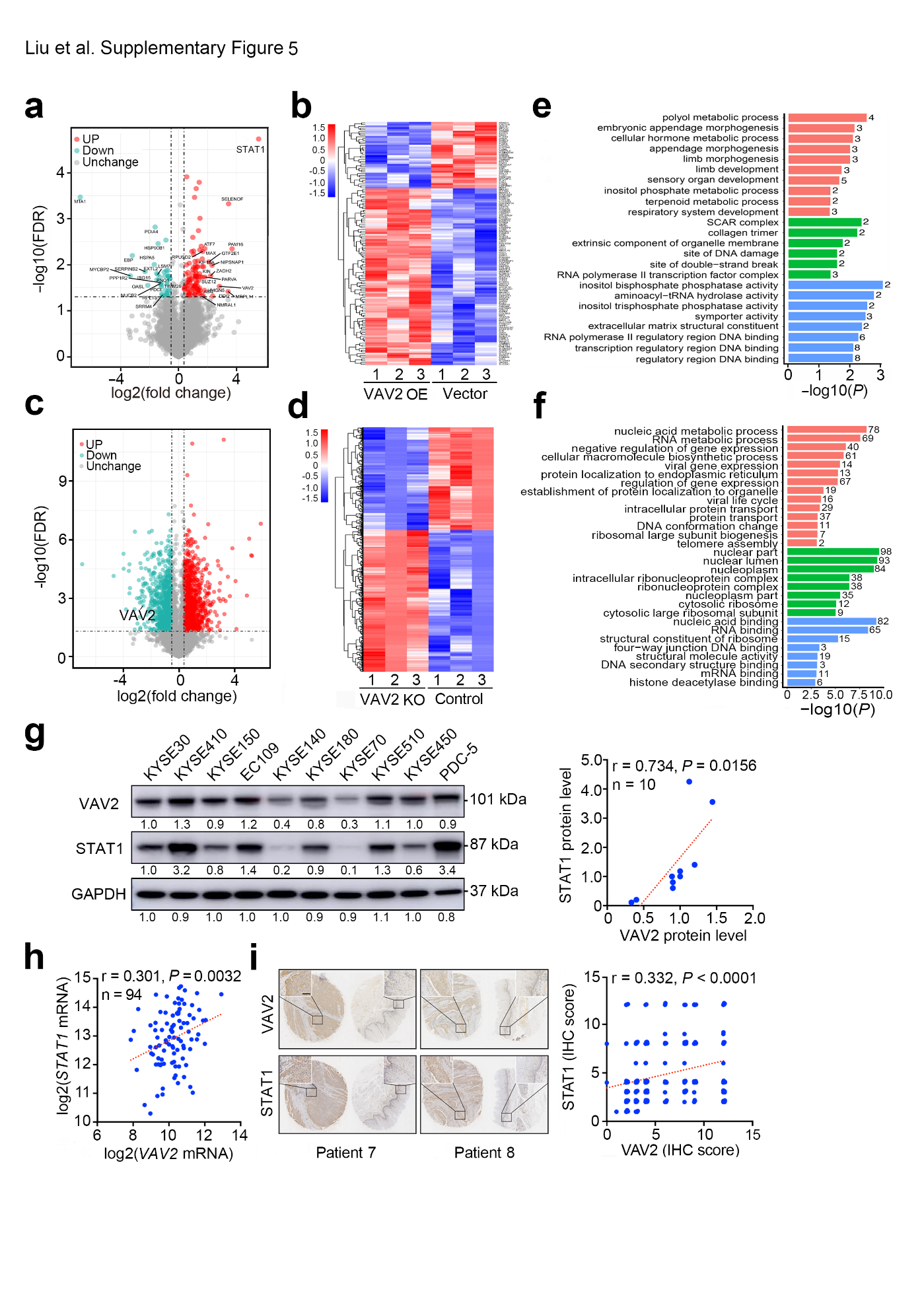


**Figure. S5. The correlation between VAV2 and STAT1 expression.**

**a**−**f** Volcano plots, Heatmaps and pathways of proteins regulated by VAV2 in cells with VAV2 OE (**a**, **b**, and **e**) or VAV2 KO (**c**, **d**, and **f**). Red, upregulated proteins (fold change > 1.3 and *P* < 0.05); green, downregulated proteins (fold change < 0.7 and *P* < 0.05) in Volcano plots. **g** Western blot analysis of the relationship between VAV2 and STAT1 in different ESCC cell lines. *Left panel* is representative images of Western blot and *Right panel* shows the spearman correlation of VAV2 and STAT1 in 10 ESCC cell lines. **h** Spearman correlation of *STAT1* and *VAV2* mRNA levels in the TCGA ESCC samples. **i** Spearman correlation of VAV2 and STAT1 protein levels in ESCC (n = 240) determined by immunohistochemical staining (IHC) score. *Left panel*, tissue arrays of VAV2 and STAT1 IHC staining. Scale bar, 100 μm. *Right panel*, Spearman correlation of VAV2 and STAT1 proteins.


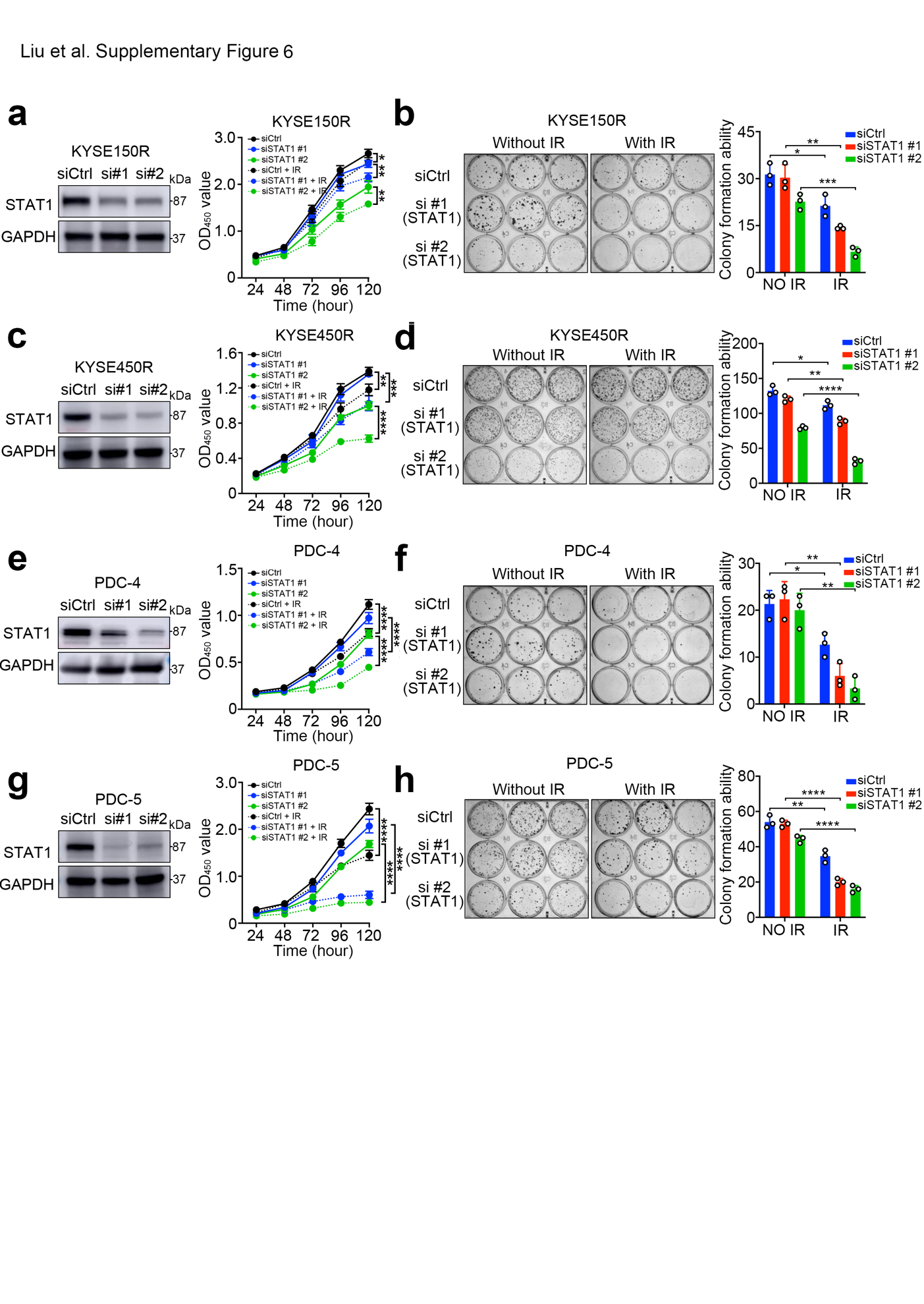


**Figure S6. Silencing STAT1 increases ESCC radiosensitivity.**

**a−g** Silencing STAT1 significantly increased the radiosensitivity of radioresistant KYSE150R (**a, b**), KYSE450R (**c, d**), PDC-4 (**e, f**) and PDC-5 (**g**, **h**) cells to irradiation (IR). Shown are cell growth (**a**, **c**, **e**, and **g**) or colony formation ability (**b**, **d**, **f**, and **h**) of cells after IR (4 Gy for cell growth and 2 Gy for colony formation, respectively). *Left panes* of **a**, **c**, **e**, and **g** show the efficiency of STAT1 silence by siRNA and *right panes* show cell proliferation curve detected by CCK-8 assays. *Left panels* **b**, **d**, **f**, and **h** show representative colony images and *right panels* show the statistics.


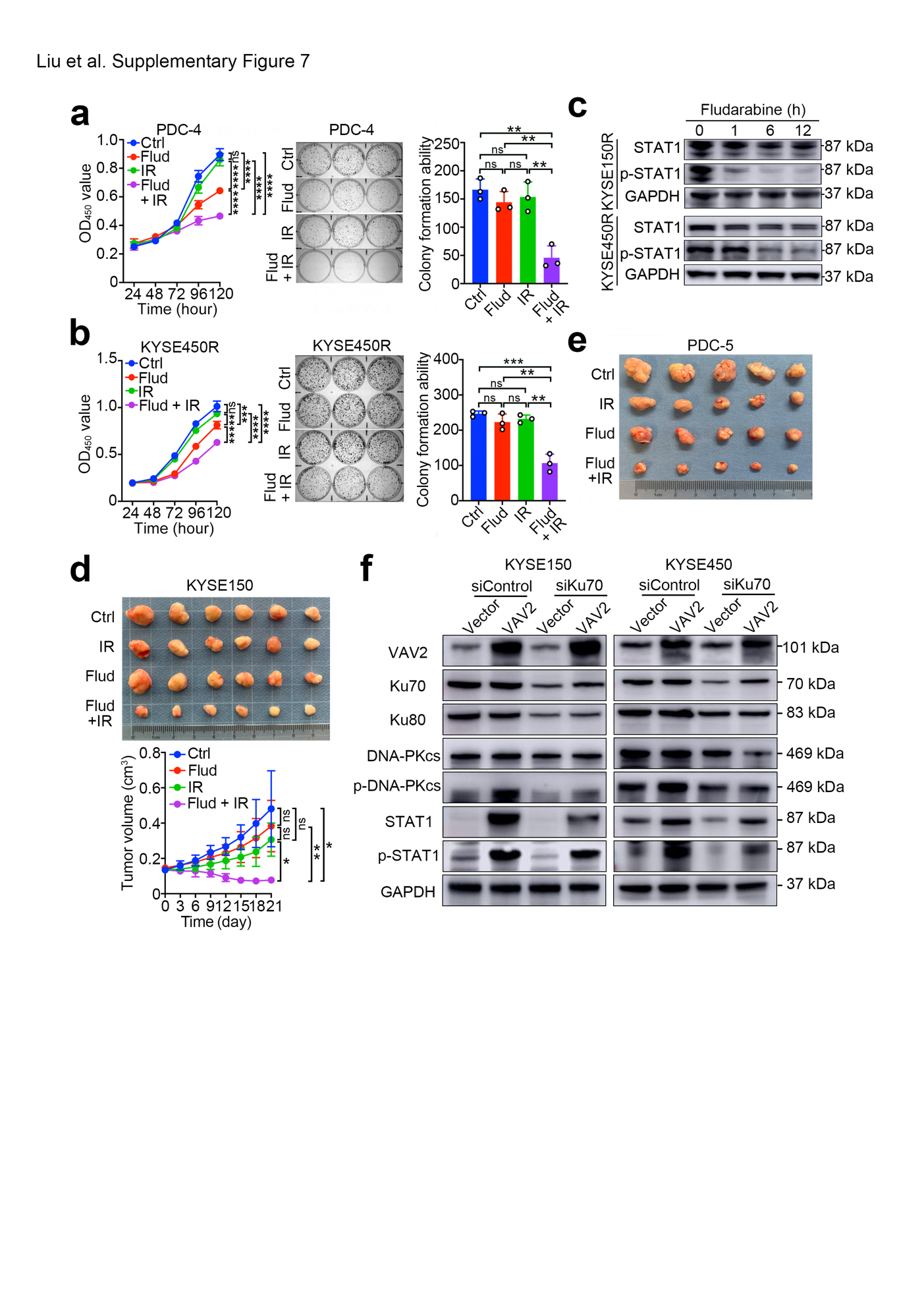


Figure S7. Additional figures related to Figure 5.

**a**, **b** STAT1 inhibitor Fludarabine significantly enhanced the radiosensitivity of radioresistant ESCC cells to IR in vitro. Shown are inhibitory effects of IR (2 Gy), Fludarabine (0.1 and 0.05 μM for cell growth or colony formation assays, respectively) or combination of IR and Fludarabine on cell growth detected by CCK-8 assays (*left panel*) and colony formation (*right panels*) of PDC-4 (**a**) and KYSE450R (**b**). Data are mean ± SEM from 3 experiments and each had 3 replications. **, *P* < 0.01; ***, *P* < 0.001; ****, *P* < 0.0001 and ns, not significant of Student’s *t*-test. **c** Western blot analysis of STAT1 and phosph-STAT1 in radioresistant ESCC cells treated with Fludarabine (0.1 μM) for different time. **d** STAT1 inhibitor Fludarabine (40 mg/kg) significantly enhanced the radiosensitivity of mouse xenografts derived from KYSE150 with VAV2 overexpression to IR (10 Gy). *Left panel* shows tumor from each mouse in different groups at the end of the experiment and *right panel* shows curves of tumor growth overtime. Data are mean ± SEM (n = 6). *, *P* < 0.05; **, *P* < 0.01 and ns, not significant of Student’s *t*-test. See methods for Fludarabine and IR treatment. **e** STAT1 inhibitor Fludarabine (40 mg/kg) significantly enhanced the radiosensitivity of mouse xenografts derived from radioresistant PDC-5 to IR (10 Gy). Shown are tumor from each mouse in different groups at the end of the experiment. **f** Western blot analysis of relevant protein levels in KYSE150 and KYSE450 cells with VAV2 overexpression and Ku70 depletion.


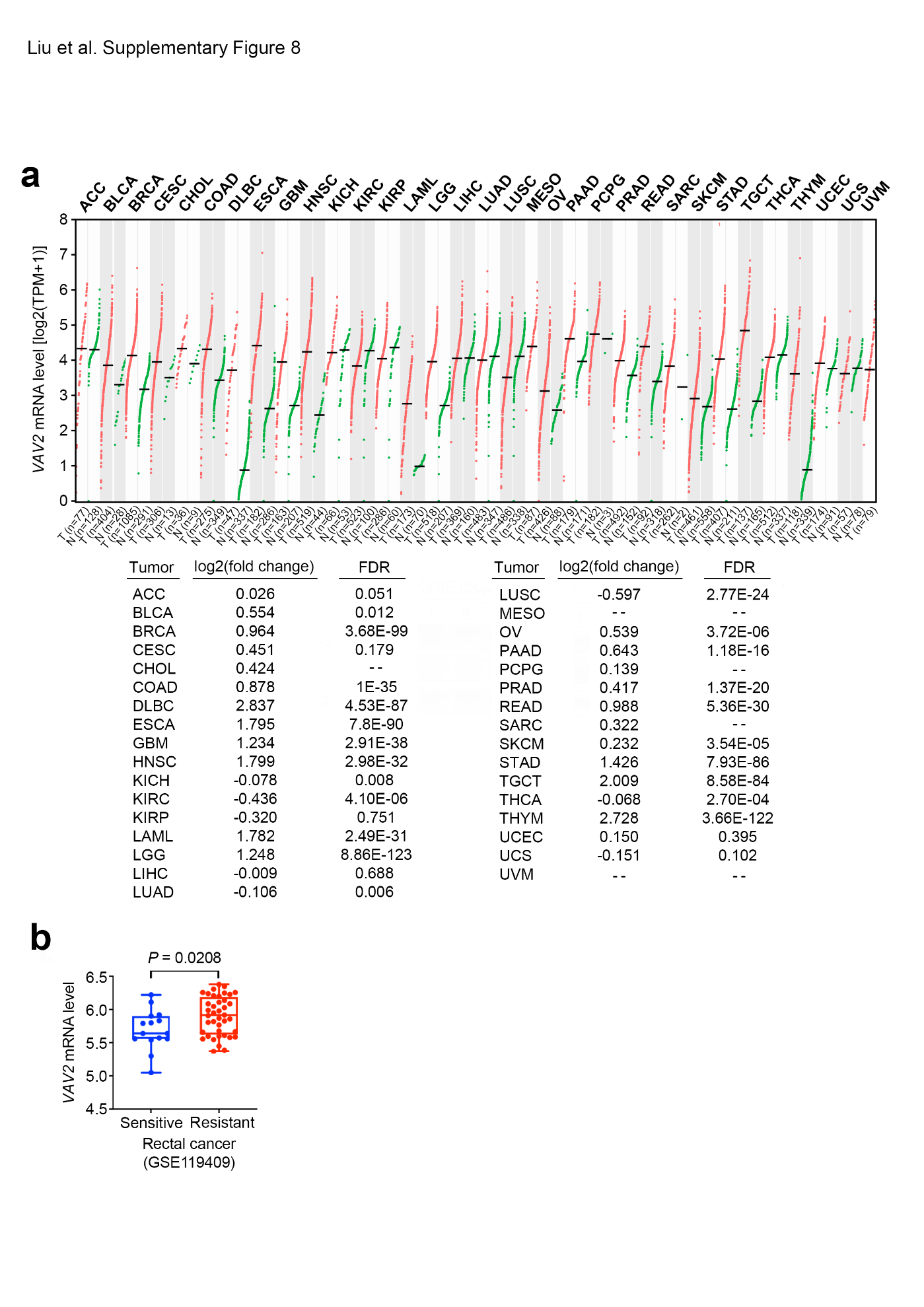


Figure S8. Correlation of VAV2 expression levels and sensitivity to radiotherapy in other types of cancer derived in public database.

**a** Differential VAV2 expression levels in tumor of 33 types of cancer and normal tissues reported in the TCGA database using the gene expression profiling interactive analysis. ACCA: adrenocortical carcinoma; BLCA: bladder urothelial carcinoma; BRCA: breast invasive carcinoma; CESC: cervical squamous cell carcinoma and endocervical adenocarcinoma; CHOL: cholangial carcinoma; COAD: colon adenocarcinoma; DLBC: lymphoid neoplasm diffuse large B-cell lymphoma; ESCA: esophageal carcinoma; GBM: glioblastoma multiforme; HNSC: head and neck squamous cell carcinoma; KICH: kidney chromophobe; KIRC: kidney renal clear cell carcinoma; KIRP: kidney renal papillary cell carcinoma; LAML: acute myeloid leukemia; LGG: brain lower grade glioma; LIHC: liver hepatocellular carcinoma; LUAD: lung adenocarcinoma; LUSC: lung squamous cell carcinoma; MESO: mesothelioma; OV: ovarian serous cystadenocarcinoma; PAAD: pancreatic adenocarcinoma; PCPG: pheochromocytoma and paraganglioma; PRAD: prostate adenocarcinoma; READ: rectum adenocarcinoma; SARC: sarcoma; SKCM: skin cutaneous melanoma; STAD: stomach adenocarcinoma; TGCT: testicular germ cell tumors; THCA: thyroid carcinoma; THYM: thymoma; UCEC: uterine corpus endometrial carcinoma; UCS: uterine carcinosarcoma; UVM: uveal melanoma. **b** The sensitivity of rectal cancer to radiotherapy by *VAV2* mRNA level in tumor. Data from the Gene Expression Omnibus database (GSE119409).

Data S1 (Separate file).

**Supplementary Table 1.** The measurements of PDX in mice.

**Supplementary Table 2.** Characteristics of 245 patients with esophageal squamous-cell carcinoma in this study.

**Supplementary Table 3.** The RNA-sequencing results of KYSE150 cells with VAV2 overexpression or knockout.

**Supplementary Table 4.** Mass spectrometry analysis identified proteins potentially associated with VAV2 in ESCC cells.

**Supplementary Table 5.** Protein level change in KYSE150 cells with VAV2 overexpression or knockout related to the corresponding control.

**Supplementary Table 6.** Characteristics of 31 patients with esophageal squamous-cell carcinoma accepted neoadjuvant chemoradiotherapy before surgery in this study.

**Supplementary Table 7.** VAV2 mRNA levels in different types of cancer of TCGA patients received radiotherapy.

**Supplementary Table 8.** Small interfering RNA sequences used in this study.

**Supplementary Table 9.** Primers used for plasmid construction or qRT-PCR analysis.
